# Probing Ce- and Zr-Fumarate Metal–Organic Framework Formation in Aqueous Solutions with In Situ Raman Spectroscopy and Synchrotron X-ray Diffraction

**DOI:** 10.1021/acsomega.4c05125

**Published:** 2024-10-21

**Authors:** Sofiia Bercha, Simmy Rathod, Olena Zavorotynska, Sachin Maruti Chavan

**Affiliations:** 1 Department of Mathematics and Physics, University of Stavanger, P.O. Box 8600, Stavanger NO-4036, Norway; 2 Department of Chemistry, Bioscience and Environmental Engineering, University of Stavanger, P.O. Box 8600, Stavanger NO-4036, Norway

## Abstract

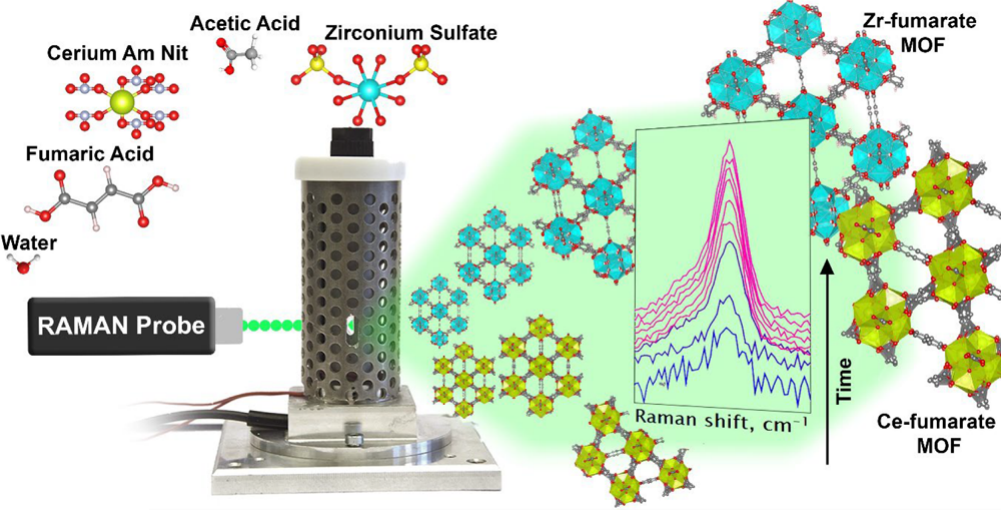

The synthesis of Ce-fumarate and Zr-fumarate metal–organic framework (MOF) is monitored for the first time with in situ Raman spectroscopy in custom-built solvothermal reactors. Several synthesis conditions were explored for Ce-fumarate at room temperature. The use of the method for high-temperature synthesis of Zr-fumarate is also demonstrated. In situ Raman monitoring provided insights into both the solution and crystalline phases of the reaction medium, revealing the dynamic interplay among precursors, modulators, and the forming MOF structure. The reaction kinetics was determined by following the characteristic peak at 1666 cm^–1^. The conversion was in good agreement with the reaction kinetics determined via in situ synchrotron powder diffraction. The resulting MOF products were further characterized using ex situ X-ray powder diffraction, scanning electron microscopy, thermogravimetry, and surface area measurements. This study demonstrates a simple and industrially scalable method for monitoring MOF synthesis in situ, which can provide insights into the stages and mechanisms of formation of MOFs and other compounds.

## 1 Introduction

Metal–organic frameworks (MOFs) are a rapidly growing class of porous materials. They have gained significant interest due to their potential applications in various fields. This includes catalysis, gas storage and separation, metal recovery, and biosensing to name few.^1−3^ Among the various reported MOFs, Zr-based compounds have excellent chemical, thermal and mechanical stability, and rigidity in harsh conditions,^4,5^ whereas Ce-based ones provide excellent redox activity that is essential in a wide area of applications, such as, for example, oxidative and photocatalysis, sensing, and so forth.^6,7^

UiO-66 is a trailblazing MOF with Zr-based oxo-cluster as the building block.^8^ It has a large, square-symmetric pore structure with a pore size of about 10 Å. Since its initial report, UiO-66 has inspired the synthesis of a substantial number of isostructural MOFs with different linkers and alternative metals.^9^ This influence extends to Ce-based isostructural MOFs, wherein cerium, akin to zirconium, demonstrates the capacity to form analogous clusters.

Understanding the mechanisms of MOF formation at the early stages of synthesis is fundamental for achieving an MOF product with the desired properties. Studying MOF formation in real time and in situ is crucial for gaining insights into the reaction kinetics, nature of key intermediates, and effect of the synthesis parameters on the reaction outcome. This ultimately enables precise control and development of MOFs with properties desired for various applications.^10^

Several experimental techniques have been utilized to follow the MOF formation in situ.^11−20^ Most typically, in situ X-ray diffraction (XRD) and X-ray total scattering with pair distribution function (PDF) analysis techniques have been employed. The in situ XRD is suitable for monitoring the formation of crystalline reaction products. For example, Zahn et al.^13^ followed the formation of Zr-fumarate during water- and DMF-based syntheses modulated with formic acid. They discovered that the reaction temperature of the water-based synthesis should be around 43 °C because the kinetics was too fast at higher temperatures to be effectively monitored with in situ XRD (at 30 s time resolution). Authors have also shown that the reaction rate exhibits an inverse relationship with the formic acid concentration. Lower concentrations of formic acid promote a faster reaction rate, whereas higher concentrations lead to a slower reaction rate and delayed crystallization. These results support the prevalence of the coordination modulation mechanism instead of deprotonation modulation, as the latter would result in shorter induction times by increasing the amount of the modulator.

Shearan et al. investigated crystallization of perfluorinated Ce(IV)-based MOFs with the in situ XRD.^16^ They have shown that without any modulator, crystallization was complete within a few seconds of Ce(IV) introduction into the reaction vessel. This kinetics was too fast to be followed with satisfactory time resolution. They have used the combination of the protonation and coordination modulators (nitric and acetic acids, respectively) to slow the reaction. Even with the modulators, the synthesis of Ce-UiO-66 was completed in only 5 min, with the nucleation phase of the synthesis being instantaneous. Another report on the in situ PXRD investigation of the formation of Ce(IV)-DUT-67-PZDC^14^ has shown that the crystallization of the MOF occurs immediately upon addition of Ce(IV) to the reaction mixture.

XRD is not well-suited to probe the earliest stages of MOF synthesis, such as dissolution of the metal precursor, cluster formation, and nucleation, as it is insensitive to subnanometer crystallites and is blind to the presence of noncrystalline phases.^18^ The pair distribution function (PDF) analysis technique provides information about the average interatomic distances within a material and allows extraction of structural information also from amorphous materials, short-range-order structures, as well as long-range-order crystalline particles. Therefore, the PDF technique is widely used to monitor the nucleation and growth of materials in situ.^19,20^ However, it is not sensitive to the chemical information during the synthesis reaction, that is, direct observation of chemical reactions and formation steps within the solvent. Vibrational spectroscopy techniques, on the other hand, can observe chemical reactions in the solvent prior to the MOF crystallization. Additionally, Raman scattering is a noninvasive, cost-effective, and readily scalable analytical technique well suitable for studies of water solutions.

Simultaneous in situ Raman scattering, Fourier transform infrared spectroscopy (FTIR), and turbidity measurements were systematically employed for the studies of the formation mechanism of MOFs by Embrechts et al.^17,21^ Using the Raman spectroscopy technique, the authors were able to describe the whole process of formation from the prenucleation building unit (PNBU) to MOF “nuclei” and to MOF crystallite formation of the MIL-53(Al) MOF. They have shown that MOF formation can be monitored by the integrated area of the characteristic Raman shift. To maximize the surface area, framework stability, and crystallinity, solvothermal synthesis should be stopped upon the detected completion of the MOF.

Another study by Chong et al.^22^ implements in situ Raman and THz Raman spectroscopy to follow the formation of the MIL-140A(Ce). Authors show that kinetic curves of the crystallization can be constructed by monitoring of the characteristic Raman peak at 762 cm^–1^ corresponding to the C–F bond of the tetrafluoroterephthalate ligand. Their results are comparable with the kinetic studies by PXRD on similar systems.

As far as we are aware, only two in situ FTIR studies that followed the modulated synthesis of Zr-fumarate MOF in DMF and water was reported by Ren et al.^12,15^ The authors have concluded that the modulator (formic acid) in the DMF- and water-based syntheses plays different roles, being a deprotonation modulator in the water-based synthesis and a coordination modulator in the DMF solvent. Water-based synthesis of Zr-fumarate resulted in a structure with fewer defects and smaller pore size.

**Figure 1:**
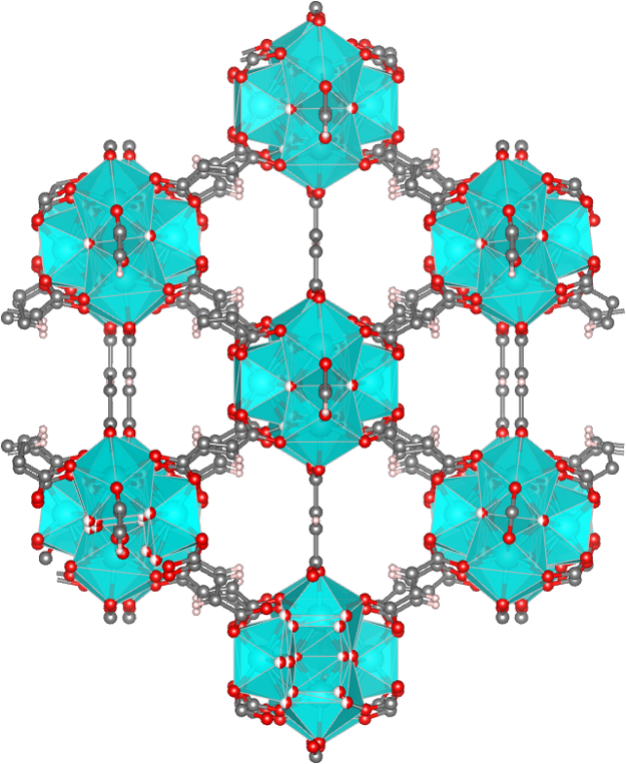
Zr-fumarate framework structure. Zr oxo-clusters are shown as cyan polyhedra and organic linkers in gray (carbon atoms). Red spheres denote oxygen atoms.

Fumarate-based MOFs (Figure 1) have applicability in various domains, from battery production,^23^ energy conversion,^24^ pollutant remover,^25^ to usage in bio- and medical applications,^26^ as well as water harvesting.^27^ Fumaric acid (HO_2_CCH=CHCO_2_H) is a biologically occurring molecule and is used as a common food additive.^28^ It is one of the nontoxic, low cost, and water-soluble linkers. MOF based on the fumaric acid linker was the first MOF upscaled on an industrial scale reactor by BASF.^29^ The simplicity of the fumarate linker is advantageous when employing the in situ Raman technique to study MOF formation for the first time.

In this paper, we present a straightforward approach for the in situ monitoring of the synthesis processes of cerium- and zirconium-fumarates. For the first time, we have used our DIY in situ reactors, inspired by design of Heidenreich et al.,^30^ for Raman measurements. The spectroscopic signal of the metal-fumarate bond was used to monitor the reaction kinetics. We have also followed the Zr and Ce cluster formation (no added linker) with in situ Raman. The MOF reaction products have been further characterized with powder diffraction, electron microscopy, thermogravimetry, and N_2_ sorption at 77 K.

## 2 Methods

### 2.1 In Situ Reaction Cell and Raman Probe

**Figure 2:**
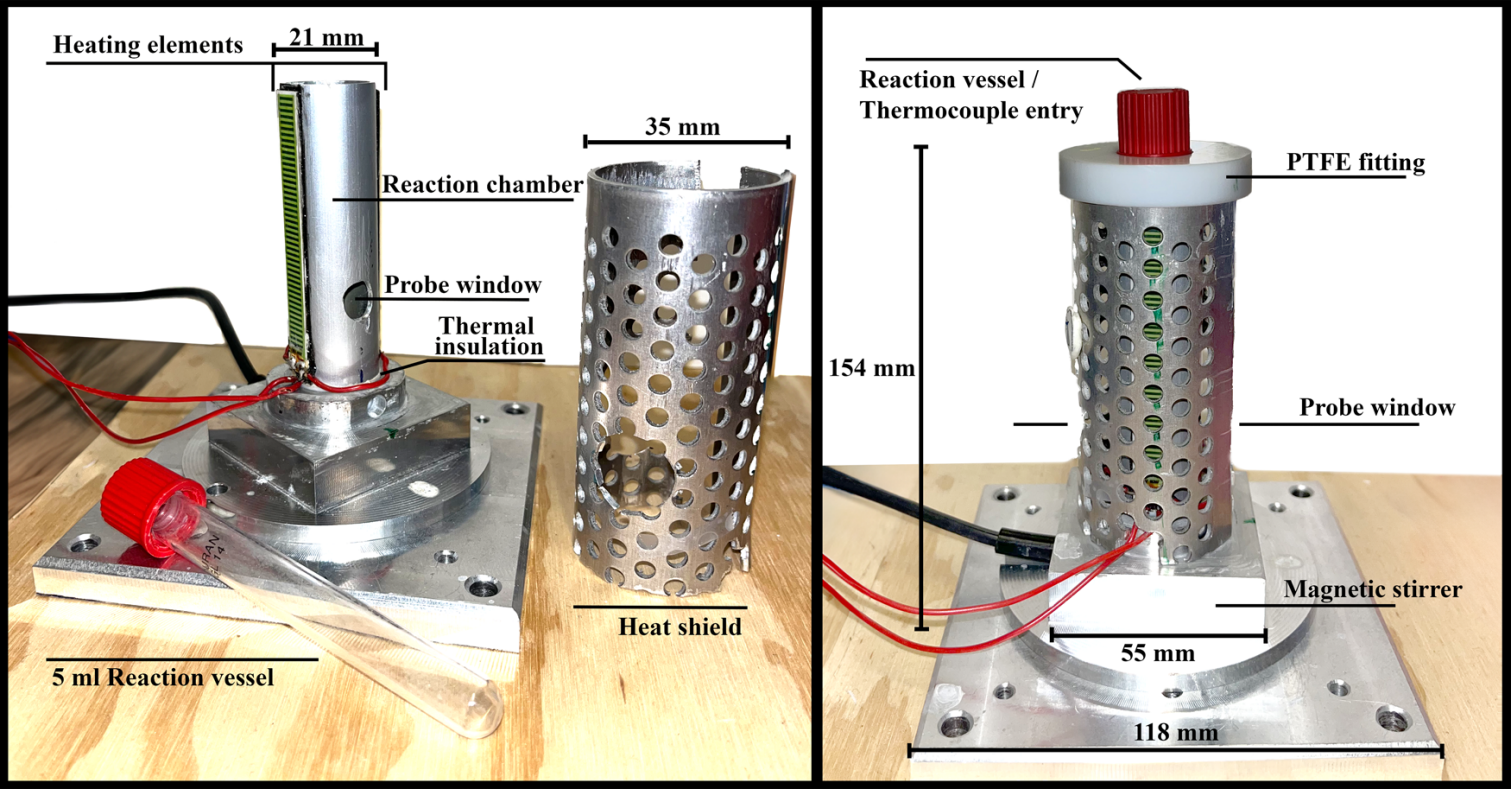
Aluminum HT in situ reactor.

Room-temperature (RT) syntheses of Ce-fumarates were performed in the 3D-printed plastic reactor (Figure S1). The high temperature (HT) synthesis of the Zr-fumarate MOF was carried out in the aluminum analogue (Figure 2). The in situ reaction cell consists of a base plate for mounting, encasing for the magnetic stirrer^31^ with a control unit and the reaction vessel holder (standard borosilicate glass vials (*V*_max_ = 5 mL)). It has openings on both sides to enable measurements of the transition geometry. The RT modification of the cell was 3D printed using common polylactic acid (PLA) filament on an Original Prusa i3MK3S+ 3D printer.^32^ The HT cell is suitable for the reactions at <140 °C. The resistive heating system is attached to the vessel holder (anodized aluminum tube) and consists of two thick film heaters^33^ connected in series. The reaction vessel is sealed with the Bohlender screw cap^34^ with the embedded K-type thermocouple to monitor the temperature inside the reactor.

The MOF syntheses were monitored by the Raman fiber optic probe with 532 nm wavelength attached to the Renishaw inVia confocal Raman microscope. The nominal power of the laser was 50 mW. The Raman experiments were performed in the backscattering geometry using the collection lens with a 6 cm focal distance that provided the measured sample volume size of about 60 μm. Each spectrum in the in situ Raman series was collected for 60 s in the 150–3200 cm^****–****1^ range. Spectral resolution was 0.5 cm^**–**1^ as determined from calibration measurements with an internal Si standard.

The areas under the spectral bands corresponding to the metal-fumarate bond were determined using a fitting procedure in Wire5 software (see the visual example in Figure 9d) within the 1600–1800 cm^–1^ range, where the Raman mode of interest is located. A linear background was applied to this region, and the data were fitted with mixed Gaussian–Lorentzian curves. Kinetic curves were constructed by evaluating the peak area associated with the formation of the metal-fumarate bond. To address uncertainties, the χ^2^ value of the fits was combined with the instrument resolution.

### 2.2 Materials and MOF Synthesis

**Figure 3:**
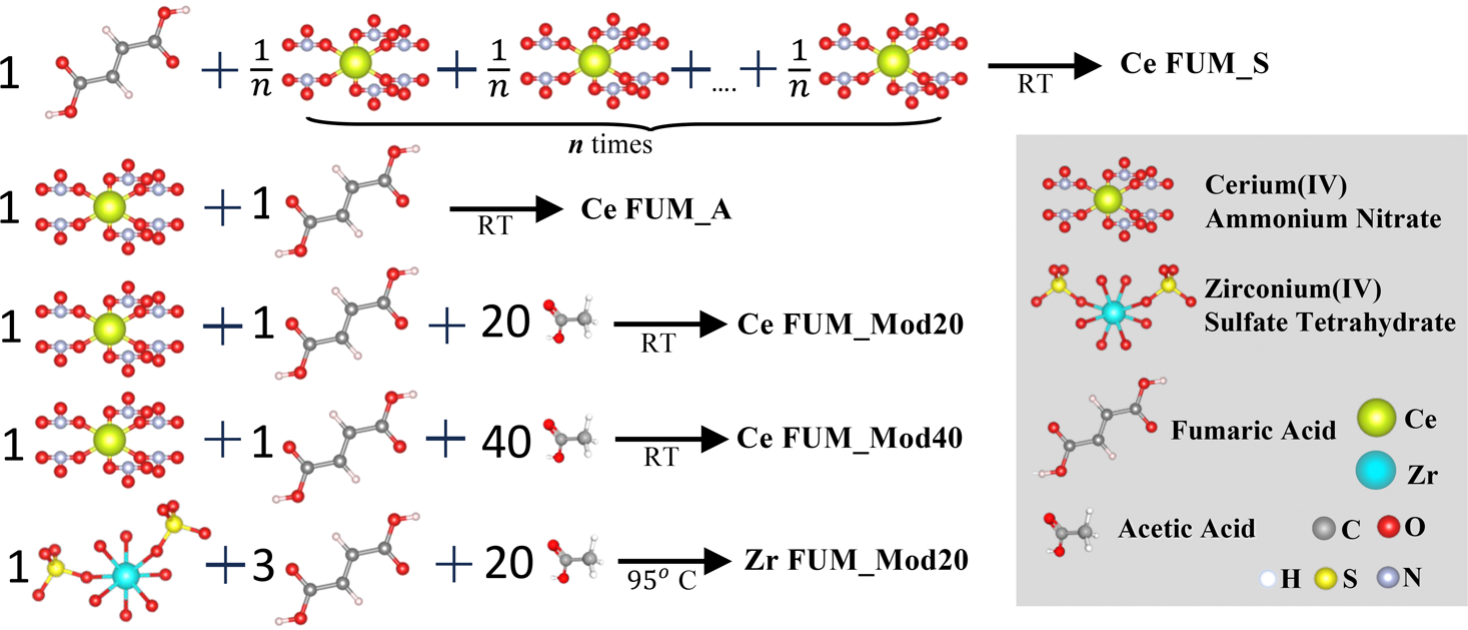
Schematics of the MOF synthesis reactions presented in this work.

This study investigates the in situ synthesis of fumarate MOFs under various conditions. We explored nonmodulated Ce-fumarate syntheses at room temperature: stepwise Ce salt addition (Ce FUM_S) and at once addition (Ce FUM_A). Additionally, modulated syntheses at room temperature were examined using different amounts of acetic acid modulators (Ce FUM_Mod20 and Ce FUM_Mod40). Finally, a modulated synthesis of Zr-fumarate at a high temperature with simultaneous addition and heating (Zr FUM_Mod20) was implemented. Schematic of the syntheses is shown on Figure 3.

Cerium(IV) ammonium nitrate (99%, Thermo Scientific), zirconium(IV) sulfate tetrahydrate (>98%, Thermo Scientific), glacial acetic acid (100%, Supelco), and fumaric acid (>99%, Sigma-Aldrich) were used for the syntheses without further purification. Reaction stoichiometries and reagent quantities are given in the Supporting Information (Table S1).

### 2.3 In Situ Synchrotron Powder X-ray Diffraction (PXRD) Measurements

The in situ PXRD data were measured at the BALDER beamline of MAX-IV synchrotron facility in Sweden with the capability of recording high-quality alternate XAS and PXRD data at few second resolution.^35^ The reactions were performed in the same custom-built reactors described above but in transmission geometry. Data collection typically commenced within 2–3 min of preparing the mixture at room temperature. Measurements were acquired in 2 s intervals, with a 2 s gap between each. The beam size focused on the sample was 70 × 80 μm. The PXRD data were obtained using a wavelength of 0.7293 Å (17000 eV) and the 2D detector EIGER 1 M (1030 × 1065 pixels, continuous readout: 1000 fps).

The Scherrer analysis was applied to the strongest peak recorded in the in situ PXRD series to evaluate crystallite growth during synthesis. The peak was fitted by using a linear background and a Gaussian function. The standard error was included in the analysis to account for the measurement uncertainties.

### 2.4 Ex Situ Characterization

The synthesized powders were first isolated by centrifugation and repeatedly washed with water. For further purification, Ce_FUM_S and Zr_FUM_Mod20 were washed with acetone and ethanol, respectively. Finally, the precipitates were dried overnight at 50 °C.

Ex situ powder X-ray diffraction (PXRD) using a Bruker D8 Advance instrument equipped with a Cu source (λ = 0.15418 nm) provided insights into the crystalline structure. Samples were prepared as suspensions on silicon wafers and measured in Bragg–Brentano geometry (40 kV, 25 mA) using a continuous scan mode (4–70° 2θ range, step size 0.01°, 80 s per step).

Scanning electron microscopy (SEM) images were obtained on a Zeiss SUPRA 35VP field-emission SEM instrument equipped with energy-dispersive spectroscopy (EDS) for elemental analysis. To enhance the conductivity and resolution, the samples were sputtered with gold prior to imaging.

Thermogravimetric analysis (TGA) was performed using a Mettler Toledo TGA/DSC 3+ STARe System with synthetic air (25 mL/min flow) as the purge gas. Samples were heated at a rate of 5 °C/min from RT to 900 °C.

N_2_ sorption isotherms were measured on a Micromeritics TriStar II system. Prior to the measurement, Ce- and Zr-MOF samples were degassed in vacuum at 60 and 100 °C, respectively, for 24 h using a Micromeritics VacPrep 061 Sample Degas System.

Ex situ Raman spectra were acquired by using a Renishaw inVia confocal microscope equipped with a 532 nm laser. Measurements were performed at 25 mW power with an exposure time of 60 s with a spot size of ∼1.5 μm and resolution of 0.5 cm^–1^.

Proton NMR spectral acquisition was performed as follows: approximately 20 mg of MOFs underwent digestion in 1 mL of 1 M NaOH solution in D_2_O. The centrifuge tube containing the mixture was allowed to stand for 24 h to ensure complete dissolution of all organic components in the solution. Subsequently, the suspension underwent separation via centrifugation and was subjected to analysis using a Bruker Ascend 400 Hz NMR spectrometer.

Ce L_3_-edge X-ray absorption spectroscopy (XAS) measurements were performed on the Ce FUM_S and Ce FUM_A samples in transmission geometry at the beamline BM31 of the European Synchrotron Radiation Facility (ESRF). The measurements were processed using Athena software.^36^ CeO_2_ powder was used as a reference material for the energy calibration. The scan range was from 5600 to 5850 eV with an energy resolution of 0.1 eV. Several scans were averaged for each sample to improve the signal-to-noise ratio.

## 3 Results and Discussion

### 3.1 In Situ Raman Characterization of Ce-Fumarate RT Syntheses

#### 3.1.1 Ce FUM_S

**Figure 4:**
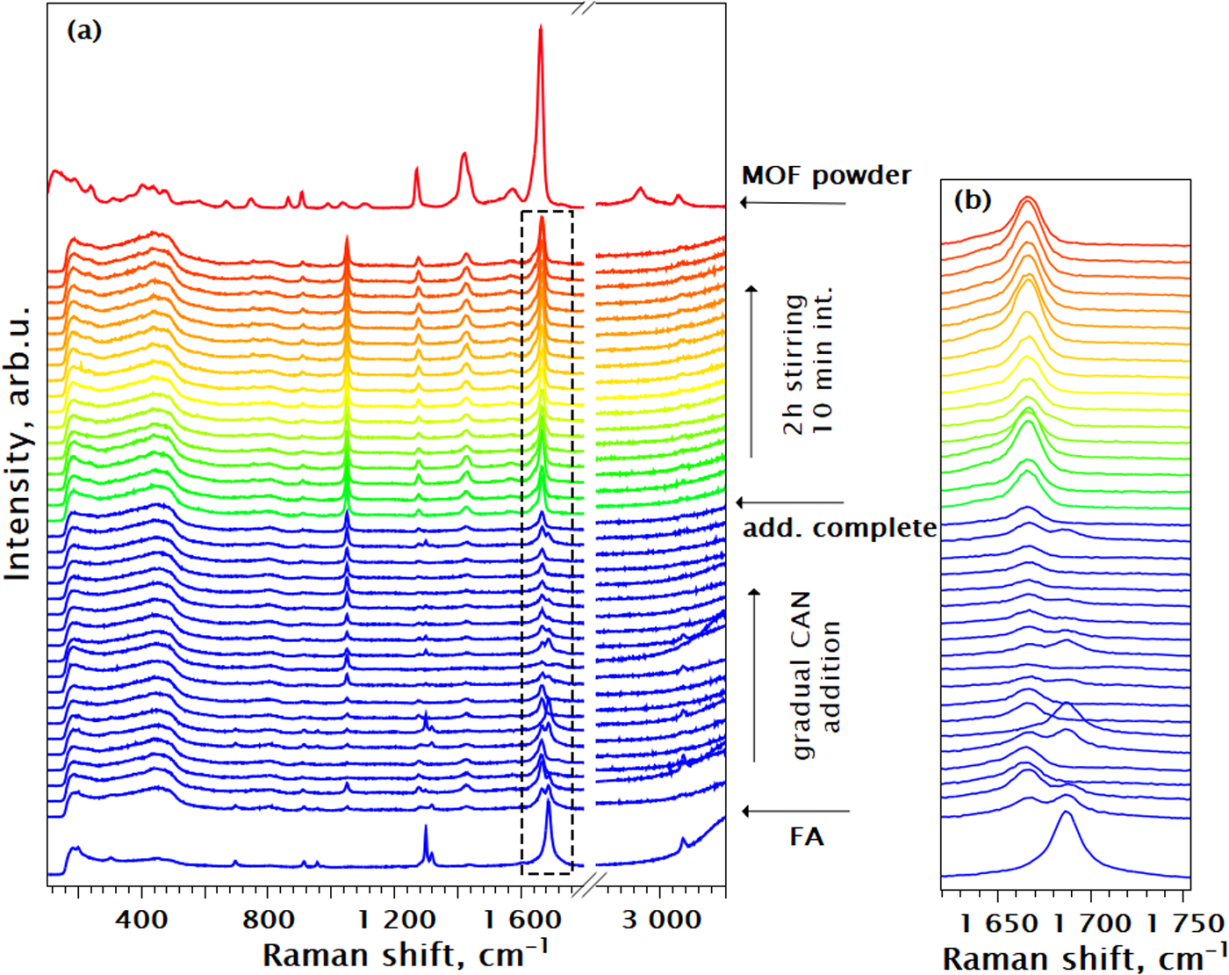
(a) In situ Raman spectra of the Ce FUM_S synthesis at RT, where Ce precursor (CAN) was added stepwise. The top orange curves provide a reference spectrum for the Ce-fumarate powder; (b) close-up of the metal-fumarate peak Raman region. Stages of stepwise synthesis are indicated with black arrows. After all the CAN was added to the solution, spectra were taken every 10 min.

Figure 4a illustrates the in situ Raman spectra following the RT synthesis of Ce-fumarate without a modulator, where Ce salt was added stepwise into the reaction vessel. The lower spectrum in blue corresponds to the water solution of fumaric acid (FA). Subsequent spectra correspond to the gradual addition of cerium ammonium nitrate (CAN) into the reaction vessel, indicated by the arrows.

The addition of the first portion of CAN causes immediate changes in the Raman spectra. The solution contains both the unreacted fumaric acid, as indicated by the characteristic C=O stretching mode at 1687 cm^–1^ and the fumarate coordinated to metal. The latter is evidenced by the appearance of a new peak at 1666 cm^–1^ that is responsible for the −O–C–O– stretching. In addition, another peak at 1050 cm^–1^ appears due to the formation of nitric acid as a result of CAN hydrolysis (see Table 1 for mode assignment). Furthermore, the changes are also observed in the ∼450 cm^–1^ region responsible for the Ce–O bonds in the Ce oxo-cluster, as compared to the Raman spectrum of reference Ce-fumarate powder, also evidencing the instantaneous formation of the inorganic building block. Upon complete addition of CAN into the reaction vessel in a stoichiometric ratio of 1:1 with fumaric acid, the evidence of unreacted fumaric acid disappeared (see Figure 4b for a magnified view), and the peak associated with nitric acid reached saturation. Any further stirring and prolonging the synthesis did not show any change in the Raman spectra.

**Table 1:** Distinct Raman Peaks during In Situ Raman Synthesis Investigation^a^

**peak/peak group Raman shift, cm**^**–1**^	**assignment**	**ref**
100–500	Ce–O, Zr–O	(39,40)
621 and lower shoulder	δ(O–S–O), sulfuric acid	(41)
889	υ(C–C), AA	(42)
913	γ(CCH), FA	(28)
1054–1037	υ(S–O), sulfuric acid	(41)
1055	υ_s_(NO_3_^–^), nitric acid	(43,44)
1280	δ (CCH), FA	(28)
1360	δ(H–C–H) bending, AA	(42)
1430	ρ(H–C–H), AA, δ (COH), FA, υ_s_(COO), metal-fumarate	(28,42,45)
1554	υ(C=C), FA	(28)
1666	υ_as_(−C=O–O), metal-fumarate	(45)
1718	υ(C=O), AA-water	(42)
1731 (with broadening)	υ(C=O), AA, and FA	(28,42)
2946	υ(CH_3_), AA	(42)
3071	υ(C–H), FA	(28)

a Abbreviations: δ, bending, υ, stretching, ρ, rocking, γ, twisting vibrations; “s” and “as”, symmetric and asymmetric modes.

These direct observations confirm that the unmodulated RT Ce-fumarate water-based synthesis results in the instantaneous metal-linker complex formation between cerium and fumaric acid in agreement with earlier studies.^16^ We have established that the metal-fumarate formation kinetics can be determined by following the −O–C–O– shift at 1666 cm^–1^, as it is the most pronounced new characteristic peak in the in situ Raman series.

#### 3.1.2 Ce FUM_A

**Figure 5:**
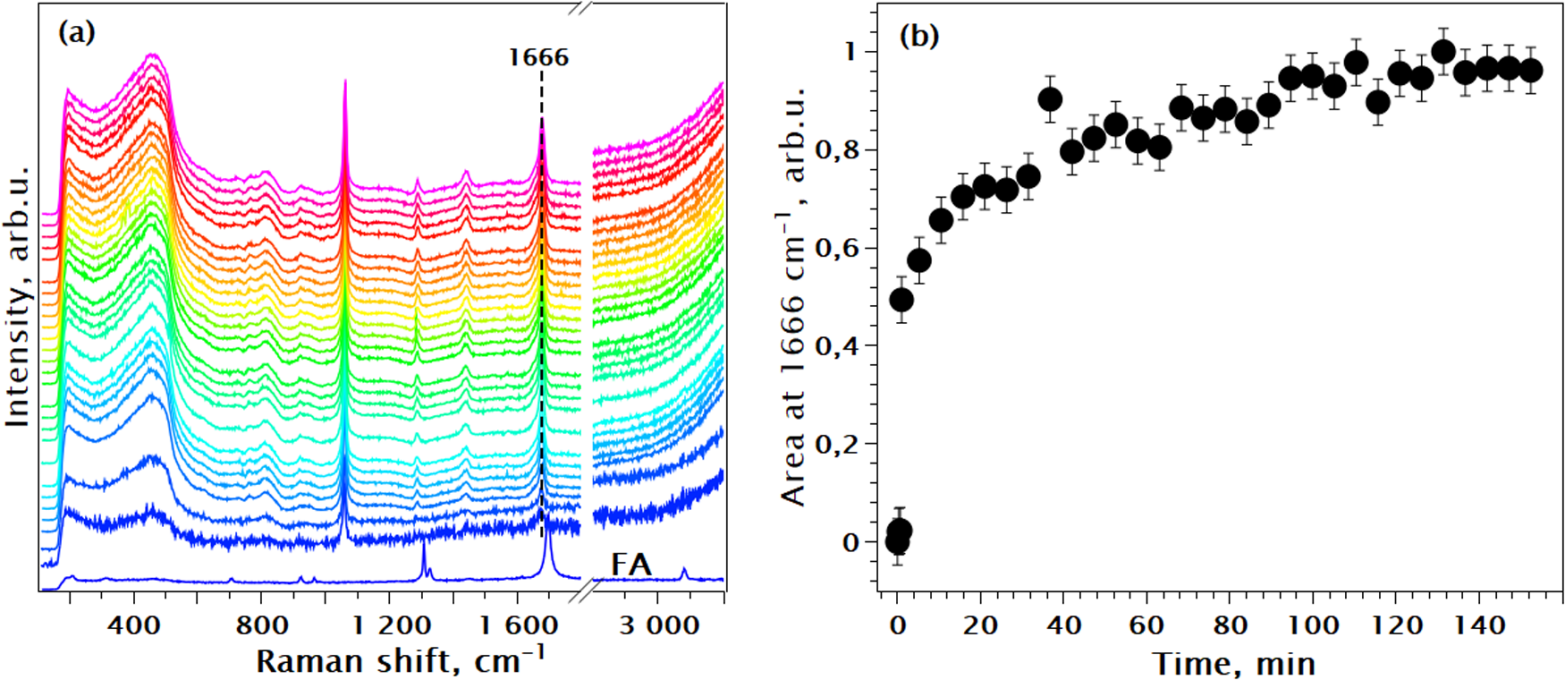
(a) In situ Raman spectra of the Ce FUM_A synthesis at RT, where all CAN was added at once. Spectra are normalized by integrated intensity. The first spectrum corresponds to the solution of fumaric acid in water before adding the precursor; (b) integrated intensity of the metal-fumarate Raman mode at 1666 cm^–1^ vs time of the reaction as the fraction of final peak area.

To strengthen the conclusions, the experiment was repeated with the addition of all CAN at once to the reaction vessel. The characteristic Raman modes remained consistent with the previous observations (Figure 5a). Moreover, this approach enabled monitoring of the continuous growth of the metal-fumarate mode over time, as depicted in Figure 5b. One can observe that the metal-fumarate bond is formed momentarily from the beginning of the reaction. Its Raman signal is starting to saturate at around 20 min slowly growing for another 100 min after that. One can also observe modifications in the 450 cm^**–**1^ region of the Ce-oxo-cluster building block coinciding with the growth of the 1666 cm^–1^ signature Raman mode.

#### 3.1.3 Ce FUM_Mod20

**Figure 6:**
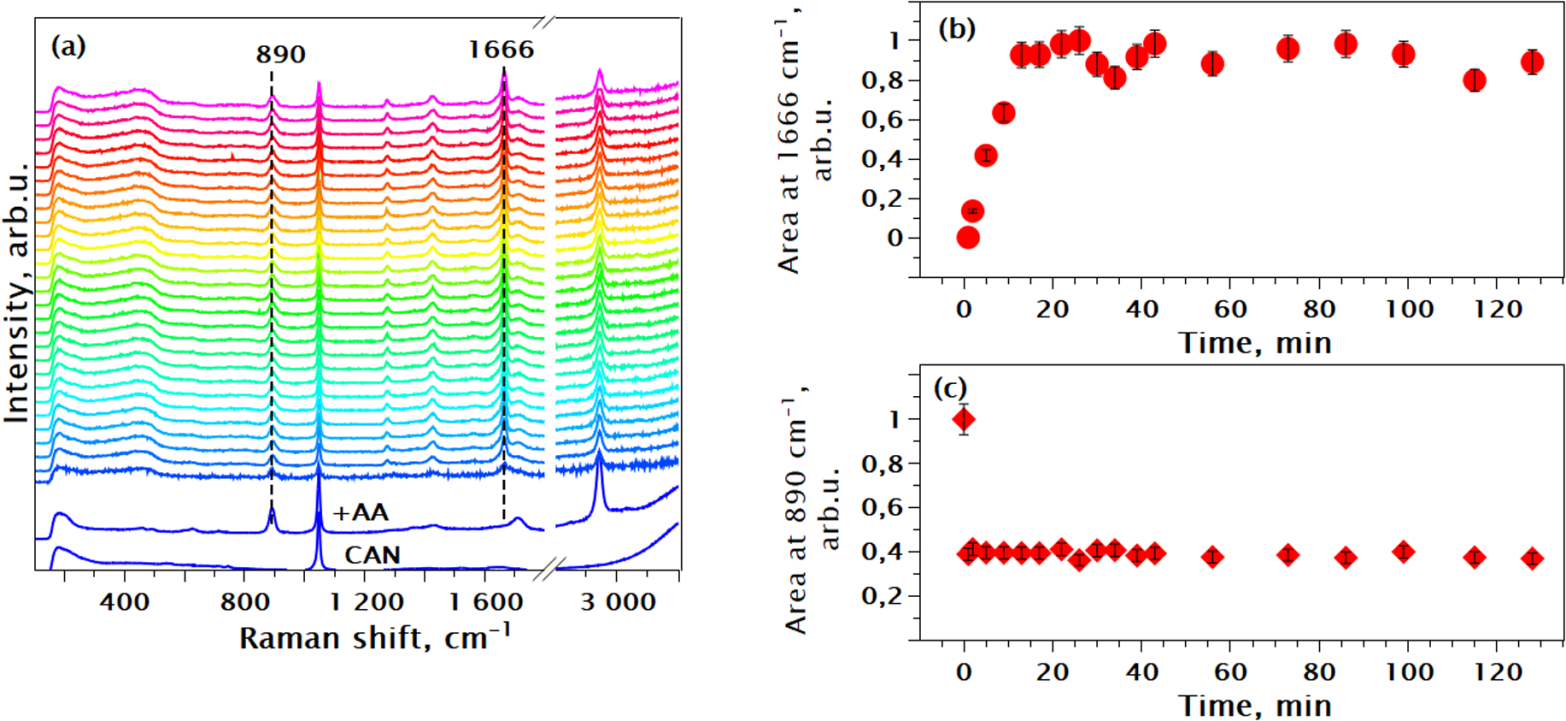
(a) In situ Raman spectra of the Ce FUM_Mod20 synthesis at RT*.* Spectra are normalized by integrated intensity. (b) Integrated intensity of the metal-fumarate Raman mode at 1666 cm^**^–^**1^ vs reaction time as the fraction of the final peak area. (c) Integrated intensity of the acetic acid mode at 890 cm^–1^ vs reaction time as the fraction of the initial peak area.

To investigate the effect of modulation, the synthesis of Ce-fumarate was repeated with the addition of 20 molar equiv of acetic acid (AA). New features due to the AA appeared in the spectra (Figure 6a and Table 1). The peak at 890 cm^–1^ (C–C stretching mode) was selected to monitor the concentration of AA due to its well-isolated position. The changes in its position would indicate the metal-acetate bond formation via the carboxylic group of the AA during modulated MOF synthesis.^37^

In Figure 6b, one can observe that the metal-fumarate bond formation is almost instant and reaches saturation within 20 min. At the same time, the 890 cm^**–**1^ mode remains intact throughout the reaction (Figure 6c) after the initial intensity drop. The position of this peak varies slightly within 888–895 cm^–1^, whereas during metal-acetate bond formation, this mode moves by about +80 cm^–1^.^38,51^ This suggests the absence of the detectable metal-acetate bond formation that in turn implies that the modulation of Ce-fumarate MOF synthesis with 20 equiv of AA does not proceed through a coordination mechanism.

To further corroborate this hypothesis, Ce-cluster formation was studied in the identical conditions but without the addition of the fumaric acid linker (Figure S2, Supporting Information). All of the observed peaks in this experiment corresponded to the combination of the acetic and nitric acid features. No shifts or new peaks were observed, and the 890 cm^–1^ feature of AA remained stable proving that the AA does not form complexes with cerium in these conditions. Indeed, when compared with the Ce-acetate Raman spectra,^38^ no evident characteristic peaks (most prominent of them at 966 cm^–1^) were detected.

Further evidence against the coordination modulation mechanism stems from the crystal grain sizes observed by SEM (Figure S8). Notably, the sizes of crystallites from the unmodulated Ce FUM_A synthesis (Figure S8b) and 20 equiv of acetic acid-modulated Ce FUM_Mod20 synthesis (Figure S8c) are the same. Further support for this statement is provided by the calculation of crystallite sizes from the in situ PXRD series, discussed in Section 3.3. This observation suggests that Ce-fumarate MOF forms instantaneously, and coordination modulation does not occur even in the presence of acetic acid as a modulator.

#### 3.1.4 Ce FUM_Mod40

**Figure 7:**
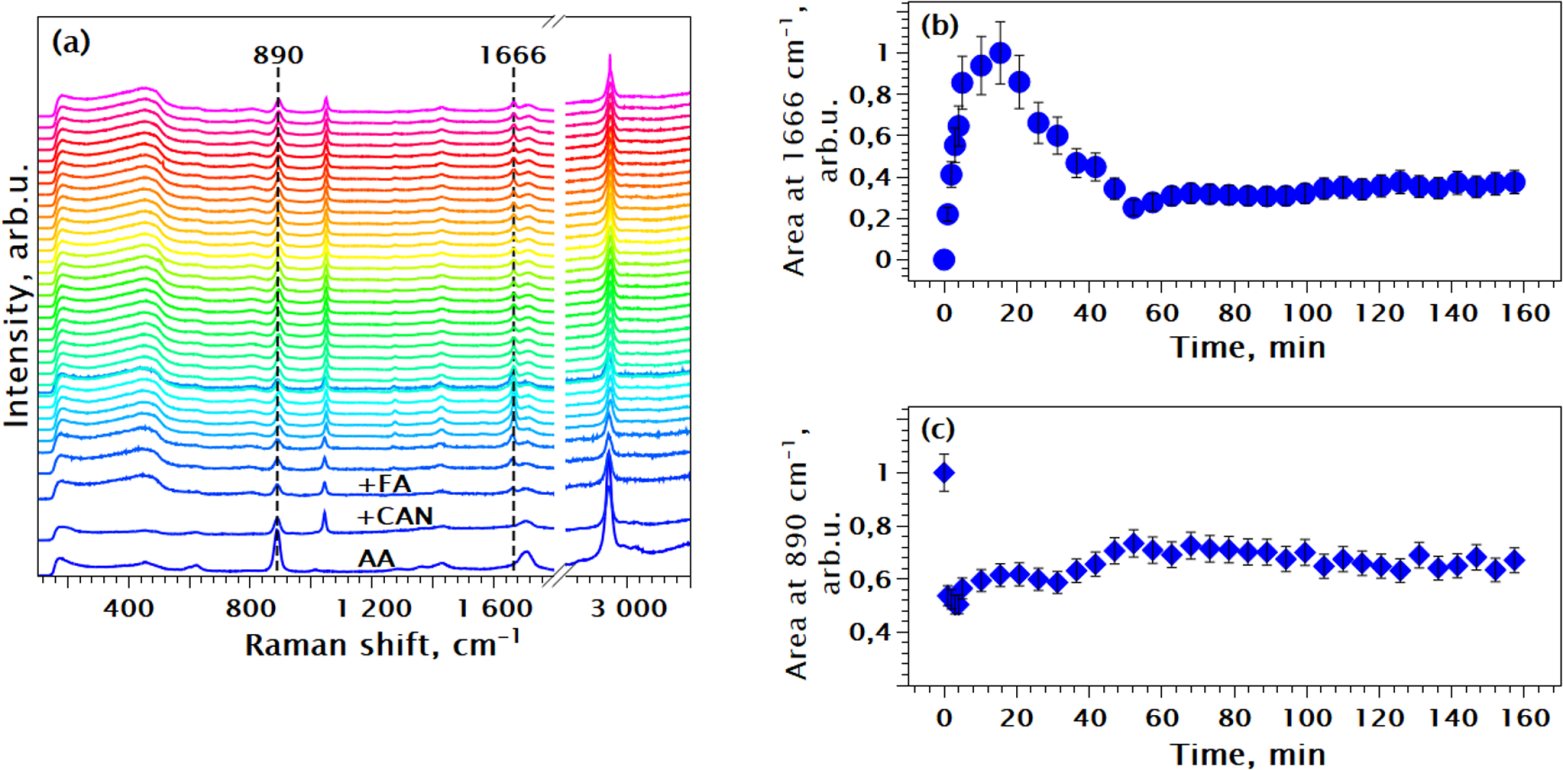
(a) In situ Raman following the Ce FUM_Mod40 synthesis at RT, data normalized by integral intensity; (b) fitted area of the metal-fumarate Raman peak at 1666 cm^**–**1^ vs time of reaction, data normalized by maximum for easier comparison; (c) fitted area of the acetic acid Raman shift at 890 cm^**–**1^ vs time of reaction, data normalized by maximum for easier comparison.

To gain deeper insight into the modulation mechanism of the water-based Ce-fumarate synthesis, the concentration of acetic acid was increased to 40 mol equiv. If the experiment demonstrates accelerated MOF formation, it will provide support for a deprotonation-driven modulation mechanism. Figure 7a shows the Raman spectra obtained during this reaction. The peak intensity at 1666 cm^–1^, exhibited a highly intriguing behavior (Figure 7b). A maximum was reached after 20 min, followed by a decrease and stabilization at around 50 min. Interestingly, the 890 cm^–1^ peak (AA) behaves differently. Its intensity declines sharply at first along with the intensities of all other peaks associated with the AA. Afterward, it gradually rises and stabilizes also at around 50 min (Figure 7c). This decline is likely due to dilution and decreased concentration of AA in the measured volume.

The observed intensity behavior of the 1666 cm^–1^ peak strongly suggests the precipitation of the MOF. This conclusion is further supported by the in situ PXRD studies discussed below. As the MOF forms, it coalesces into larger particles that precipitate out of the measurement area. By 20 min, the reaction reaches completion, leaving fewer MOF particles in the measurement volume; hence, the peak intensity declines.

An alternative explanation for the intensity decrease of the 1666 cm^–1^ peak could be exceeding the optimal reaction time. Stopping the reaction at 20 min (peak intensity maximum) might improve the MOF yield and quality based on the reported observations.^6,21^ However, this hypothesis is challenged by extending the synthesis to 24 h without evidence of MOF decomposition, as reported in previous studies.^6^ The PXRD patterns and Raman spectra of the washed and dried products (see Figure S11 in the Supporting Information) were consistent after the syntheses for 70 min, 2.5, and 24 h.

While the absence of coordination-based signatures suggests an alternative mechanism, identifying the modulation pathway (if indeed one exists) remains elusive. To explore the possibility of a deprotonation mechanism, as proposed by Ren et al. for formic acid-modulated Zr-fumarate synthesis,^15^ we conducted a simple pH-variation study, discussed below.

**Figure 8:**
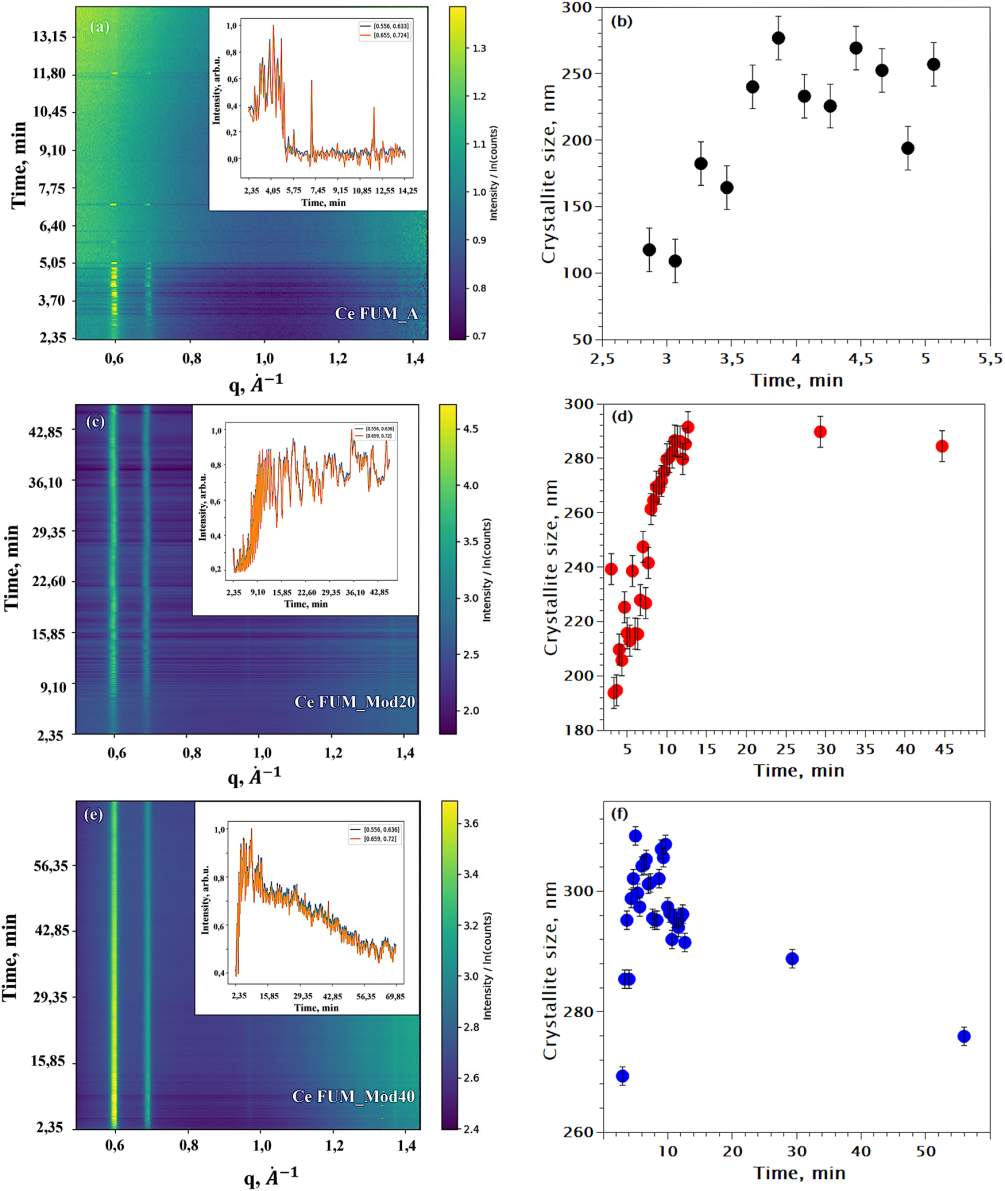
In situ PXRD data obtained during the Ce-fumarate synthesis reactions. The insets on panels (a), (c), and (e) show the time dependence of the integrated area of the first two peaks (blue and orange lines for first and second peak, respectively) after the background subtraction. (a) Ce FUM_A; (b) crystallite size changes in the Ce FUM_A synthesis; (c) Ce FUM_Mod20; (d) crystallite size changes in the Ce FUM_Mod20 synthesis; (e) Ce FUM_Mod40; (f) crystallite size changes in the Ce FUM_Mod40 synthesis.

Increasing the AA concentration did not accelerate the MOF formation reaction, nor did it lead to detectable Ce-acetate formation or changes in the crystal grain size (Figure S8 in the Supporting Information) and additional crystallite size growth (Figure 8). These observations suggest that neither of the well-established modulation mechanisms is active in these syntheses. Therefore, we propose that adding acetic acid as a modulator in the concentrations investigated has a minimal impact on the Ce-fumarate synthesis.

### 3.2 pH Study

To elucidate the role of acetic acid as a modulator in Ce-fumarate synthesis, we assessed the pH of the precursors’ water solutions (individually and combined accordingly) using pH strips (0–6 range). These ex situ pH measurements were performed immediately after mixing and approximately 30 min into reaction time. Fumaric acid’s p*K*_a_ values (4.21, 3.01) and acetic acid’s p*K*_a_ (4.76) indicate that below pH 4, only fumaric acid is monodeprotonated and below pH 3, all acids are protonated. For both Ce FUM_Mod20 and Ce FUM_Mod40, the pH remained below 3 throughout the synthesis, eliminating deprotonation modulation as a possible mechanism. Additionally, in situ Raman spectra, including those probing metal cluster formation, revealed no evidence of coordination modulation, as discussed previously.

### 3.3 In Situ Synchrotron PXRD Studies of Ce-Fumarate RT Syntheses

To support our Raman results of instantaneous MOF formation, we also performed in situ synchrotron PXRD a commonly used technique to study the MOF crystallization. The in situ PXRD analysis for Ce FUM_A, Ce FUM_Mod20, and Ce Fum_Mod40 were carried out in conditions identical to the Raman studies and in the same in situ reactors. It is clearly evident that reflections from the crystalline MOF phase appear at the very beginning of the experiment. In the Ce FUM_A synthesis (Figure 8a), the collected data exhibited disruptions/missing patterns. The signal completely disappeared within approximately 5 min, reappearing sporadically at later time points. Analysis of the crystallite size growth (Figure 8b), performed using Scherrer the approach, indicates an increase in crystallite size to approximately 300 nm within this 5 min time frame (inset in Figure 8a). Evidently, the MOF crystallites have grown, agglomerated, and precipitated from the measurement point at this time. Notably, the ex situ PXRD patterns of the washed and dried Ce FUM_A powder obtained during the in situ PXRD (15 min synthesis), and Raman experiments (2.5 h synthesis) are remarkably similar (Figure S11 in the Supporting Information).

Unlike in Ce FUM_A, in Ce FUM_Mod20, less pronounced precipitation was observed. Clear saturation of first two peaks’ intensity at around 13 min can be observed (Figure 8c) suggesting complete MOF formation. For the Ce FUM_Mod40, the mark of saturation was again 13 min (Figure 8e) followed by the decline in intensity. This decline of the signal we again assign to precipitation of the MOF product, not to loss of crystallinity or decomposition of the MOF, as the obtained washed and dried powder shows good crystallinity and is again identical with the sample obtained after the in situ Raman experiment (Figure S11 in the Supporting Information).

Figure 8b,d,f illustrates the changes in crystallite sizes throughout the reaction. It is evident that in all of the discussed syntheses, Ce-fumarate MOF crystallites grow to approximately 300 nm, and extending the reaction time beyond this point does not result in further size increase. Additionally, the increasing amounts of acetic acid lead to different crystallite sizes at the start of measurement (approximately 2.5 min into the reaction). The initial crystallite sizes are around 120 nm for Ce FUM_A, 190 nm for Ce FUM_Mod20, and 270 nm for Ce FUM_Mod40. This variation is attributed to the pH effect, which influences the growth rates of the crystallites.^2^

The observed variations in the precipitation rates of the crystalline Ce-fumarate MOFs across different synthesis procedures can be attributed to the changes in the reaction solution’s viscosity. Adding acetic acid significantly increases water viscosity,^46^ impacting particle settling behavior. In the nonmodulated Ce FUM_A synthesis, the aggregated MOF particles precipitate rapidly due to the lower viscosity. While a higher viscosity in Ce FUM_Mod40 hampers the precipitation, the moderate viscosity in Ce FUM_Mod20 created a balance between the buoyancy, drag force, and gravity that allowed MOF particles to remain within the measurement volume. This in situ PXRD experiment, unlike the in situ Raman, ensures the same measurement point for all three syntheses, strengthening the conclusion regarding viscosity’s influence compared to Raman experiments with potentially varying measurement spots.

Combining all of the above studies reinforces the conclusion that acetic acid plays a minimal or negligible role as a modulator in Ce-fumarate MOF synthesis. This finding simplifies our understanding of the Ce-fumarate synthesis pathway. Upon combining fumarate linker and CAN in the reaction vessel, rapid formation and crystallization of Ce-fumarate MOF are observed. Ce oxo-clusters form promptly in parallel with coupling to the fumaric linkers, which initiates MOF crystallization immediately. The parallel formation of Ce oxo-clusters and their coordination to the fumarate linker are evident from the Raman series (Figures 4–6), where the modifications in the ∼450^–1^ cm region (formation of Ce oxo-cluster) occur at the same time as the formation and growth of the 1666 cm^–1^ peak (linker–metal coordination). The increasing intensities of the ∼450 cm^–1^ region and 1666 cm^–1^ peak and the PXRD features in the diffraction series (Figure 8) correspond to the crystallite growth up to the size of approximately 300 nm at which they can precipitate from the measurement volumes. This phenomenon manifests as a decrease in the corresponding intensities.

### 3.4 Zr-Fumarate HT Synthesis

To comprehensively evaluate the HT reactor’s capabilities and compare kinetic data with the previous in situ studies of similar systems,^13,15^ a high-temperature modulated synthesis of Zr-fumarate MOF was undertaken.

**Figure 9:**
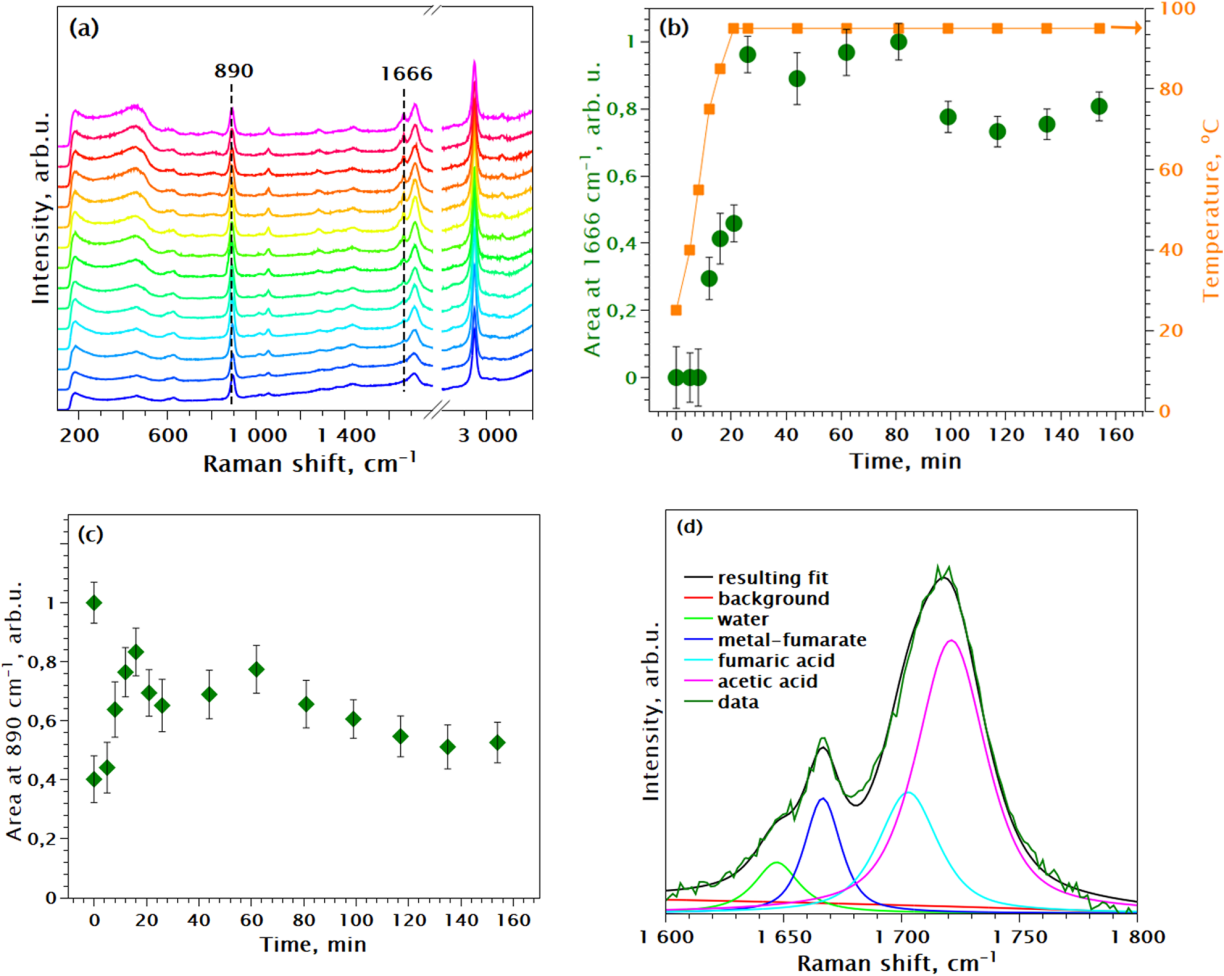
(a) In situ Raman following the Zr FUM_Mod20 synthesis at HT, data normalized by integral intensity; (b) fitted area of the metal-fumarate Raman peak at 1666 cm**^–^**^1^ vs time of reaction and temperature of the synthesis, data normalized by maximum for easier comparison; (c) fitted area of the acetic acid Raman peak at 890 cm**^–^**^1^ vs time of reaction, data normalized by maximum for easier comparison; (d) example of the peak fitting in the 1600–1800 cm**^–^**^1^ range.

Figure 9a shows the in situ Raman series following the synthesis of Zr FUM_Mod20. Figure 9b illustrates the time- and temperature-dependent behavior of the metal-fumarate bond formation peak at 1666 cm^**–**1^. The intensities were normalized to RT (see Supporting Information Section S3 for clarification). The Zr-fumarate MOF obtained through modulated synthesis exhibited Raman peaks remarkably similar to those observed in the Ce-fumarate modulated synthesis. Here, the 1055 cm^–1^ mode originates from the S–O stretching of the hydrolyzed Zr precursor. Zr(SO_4_)_2_·4H_2_O was utilized in these experiments in place of traditionally used chloride/oxychloride precursors due to the fluorescence of the chloride-based precursors with our 532 nm laser probe. Utilization of the Zr sulfate for synthesis of Zr-based MOFs was reported earlier in the studies on green synthesis and scale-up of MOF production.^47−49^

Consistent with the previous studies,^13,15^ our results reveal an induction period of approximately 10 min for the Zr-fumarate bond formation. This time coincides with the saturation of the υ(S–O) peak at 1055 cm^–1^ (at the point of the cyan curve in Figure S4), indicating complete hydrolysis of the Zr salt and its readiness to participate in MOF formation. At the same time, we also observe the formation and saturation of weak features at 1016 and 986 cm^–1^ and at 3026 and 3000 cm^–1^. The metal-fumarate formation peak (1666 cm^–1^) reaches its maximum intensity and saturates by 25 min, corresponding precisely to the time when the reaction vessel temperature reaches 95 °C. A subsequent minor decrease in the peak intensity around 90 min could be attributed to fitting uncertainties or, alternatively, product precipitation.

Simultaneously with the appearance and growths of the 1666 cm^–1^ feature, after the 10 min induction time, substantial alterations observed in the M–O cluster region (100–600 cm^–1^) serve as a distinct indicator of Zr-oxo-cluster formation upon reaching 95 °C (illustrated by the pink curve in Figure S4 in the Supporting Information). The weak features at 1016 and 986 and 3026 and 3000 cm^–1^ during this time decline in intensity.

These observations allow us to tentatively outline the steps in the reaction pathway. During the initial 10 min induction period, which involves Zr salt hydrolysis, an intermediate Zr phase is formed.^20^ This intermediate is characterized by moderate-intensity scattering around 450 cm^–1^ associated with the Zr–O core. As the reaction progresses, the peak at 1666 cm^–1^ grows, indicating the coordination of the fumarate. Simultaneously, alterations in the 450 cm^–1^ region indicate the growth of Zr oxo-clusters and the formation of the MOF.

In this synthesis, the molar ratio between the Zr salt and fumaric acid was 1:3, unlike the 1:1 ratio used in the Ce-fumarate synthesis. Whereas this excess linker leads to the presence of both reacted and unreacted fumaric acid and the features of coordinated and noncoordinated linker in the Raman spectra, Figure 9d demonstrates that the distinct peaks in the spectrum allow for straightforward integration and differentiation between them. Consequently, the kinetic curve can be constructed by monitoring the intensity changes of the same −O–C–O– mode as used previously. Alternatively, a less intense but more isolated peak at approximately 1440 cm^–1^, corresponding to the symmetrical stretching of the same −O–C–O– bond (Table 1), could be used for constructing the kinetic curve. This mode exhibits identical growth behavior to the 1666 cm^–1^ peak. Section S10 in the Supporting Information further corroborates the position of these two peaks and identifies the coordination mode in fumarate MOFs.

The integrated intensity fluctuation of the 890 cm**^–^**^1^ peak (Figure 9c) likely stems from a confluence of factors within the reaction vessel. These factors include variations in the local concentration of AA within the measurement spot, temperature-induced changes in the intensity,^50^ inherent measurement and fitting errors, and the system’s elevated background. Direct comparison of the kinetic parameters between the Zr-fumarate synthesis in this study and those reported by Zahn et al.^13^ and Ren et al.^15^ is hindered by several significant differences in their approaches. Notably, they employed different Zr salts (Zr chloride in both cases) and stronger formic acid as a modulator.

Due to the significantly lower pH resulting from the hydrolysis of Zr sulfate (generating a stronger sulfuric acid compared to the nitric acid in Ce-fumarate synthesis), both acetic and fumaric acids remain fully protonated throughout the reaction. This low pH environment effectively eliminates deprotonation modulation of the Zr-fumarate MOF, aligning with observations in the Ce-fumarate system.

## 4 Postsynthetic Characterization of the Reaction Products

**Figure 10:**
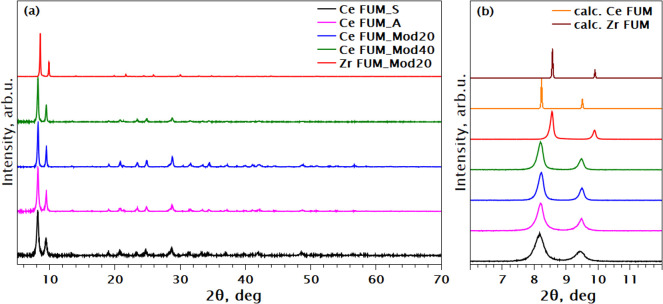
(a) PXRD of washed and dried MOFs, normalized to maximum; (b) low-angle fragment of the PXRD, with the calculated patterns of Ce FUM MOF and Zr FUM MOF for comparison.

All the reported MOF synthesis reaction result in MOF formation. PXRD of all the reaction products (Figure 10) is in agreement with the theoretically predicted and experimentally reported patterns for Zr- and Ce-fumarate MOFs.^6,51^ The observed significant shift of the Zr-based MOF to higher 2θ angles is expected due to the smaller atomic radius of Zr compared to Ce, resulting in a smaller unit cell parameter in Zr-fumarate. Lower-angle PXRD peaks for Ce-fumarate MOFs are similar except Ce FUM_Mod40 with a slight shift to lower angles, indicating a larger unit cell.

Table 2 shows the surface area measurement results of the washed and dried MOFs from N_2_ adsorption measurements and the BET calculation method. Figure S9 in the Supporting Information presents the pore size distribution in the samples. All the samples except Ce FUM_S exhibit surface areas comparable to the previously reported values.^52^ The exceptionally low surface area of Ce FUM_S is attributed to the trapped molecules introduced during the stepwise CAN addition synthesis, as discussed earlier (also refer to Section S4 in the Supporting Information).

**Table 2:** Surface Area of Washed and Dried MOFs Monitored with N_2_ Adsorption

sample	BET, m^2^ g^–1^
Ce FUM_S	254
Ce FUM_A	685
Ce FUM_Mod20	889
Ce_FUM_Mod40	857
Zr FUM_Mod20	683

Interestingly, using acetic acid in the synthesis enhances the surface area of Ce-fumarate MOFs by approximately 30% compared with the unmodulated Ce FUM_A counterpart. This effect is observed for both Ce FUM_Mod20 and Ce FUM_Mod40. However, doubling the modulator quantity (comparing Ce FUM_Mod20 and Ce FUM_Mod40) does not lead to a further increase in the surface area.

Ideal fumarate MOFs, due to their short linker length, should possess pores sized of about 0.5–0.7 nm. However, larger mesopores with an average diameter of 1.5–2 nm have been reported.^12,13,27^ Our equipment limitations prevent analysis of the lower pore size range in Figure S9 (Supporting Information). Nevertheless, mesopore sizes can be compared across samples.

Nonmodulated Ce-based MOFs (Ce FUM_S and Ce FUM_A) exhibit single mesopore sizes of 1.16 and 1.25 nm, respectively. Modulated synthesis (Ce FUM_Mod20) showed two sizes: a primary peak at 1.24 nm and a minor peak at 1.54 nm. Ce FUM_Mod40 displays three sizes: a dominant peak at 1.18 nm and weaker features at 1.55 and 1.85 nm. Zr FUM_Mod20 exhibits three distinct mesopores at 1.25 1.55, and 1.85 nm. While TGA, XRD, and the absence of acetic acid in NMR suggest no defects in this sample, the observed multiple pore sizes might indicate otherwise. For Ce-based MOFs, this size distribution further supports that modulated synthesis results in increased defect quantity and variety.

There are many reports on modulator-induced defects in MOFs, particularly UiO-66, where stronger and higher concentration modulators lead to more cluster defects.^15,53−57^ These defects can show unique PXRD peaks at lower angles. However, our samples lack such peaks, suggesting either randomly distributed or minimal cluster defects. Thermogravimetric analysis (TGA) was used to establish the amount of defects in the MOF structures; however, it cannot differentiate linker and cluster defect amounts.

Figure S7 shows TGA curves of the MOFs. Weight loss calculations for ideal Ce- and Zr-fumarate compounds are provided in the Supporting Information. By comparing these values to the TGA data, we can analyze defect levels. The Zr FUM_Mod20 sample shows no detectable defects by TGA (details in the Supporting Information and Table S2). For Ce-based samples, Ce FUM_S exhibits a significant amount of trapped moieties in its pores. We suggest that gradual salt addition during the synthesis and immediate ceria-fumarate linker bond creation led to pore formation around cerium species, encapsulating them. Proton NMR of this sample (Figure S5 in the Supporting Information) showing protons corresponding to acetone (used for washing) and an unknown moiety at 8.4 ppm in the pores supports this hypothesis. Similar TGA profiles are observed for other Ce-based samples (Figure S7). We estimate a 16% defect level, corresponding to an average of one missing linker per cluster (excluding cluster defects, see the Supporting Information). This aligns with the consistent acetic acid content in proton NMR (Figure S5, Supporting Information), which we propose caps the missing linkers for charge balance. The thermal decomposition temperatures of the synthesized Ce-based MOFs (approximately 250 °C) and the Zr-based MOF (approximately 450 °C) are in good agreement with previously reported values.^52^ Notably, the Ce FUM_Mod40 sample exhibits a slightly higher decomposition temperature compared to those of the other Ce-based materials.

**Figure 11:**
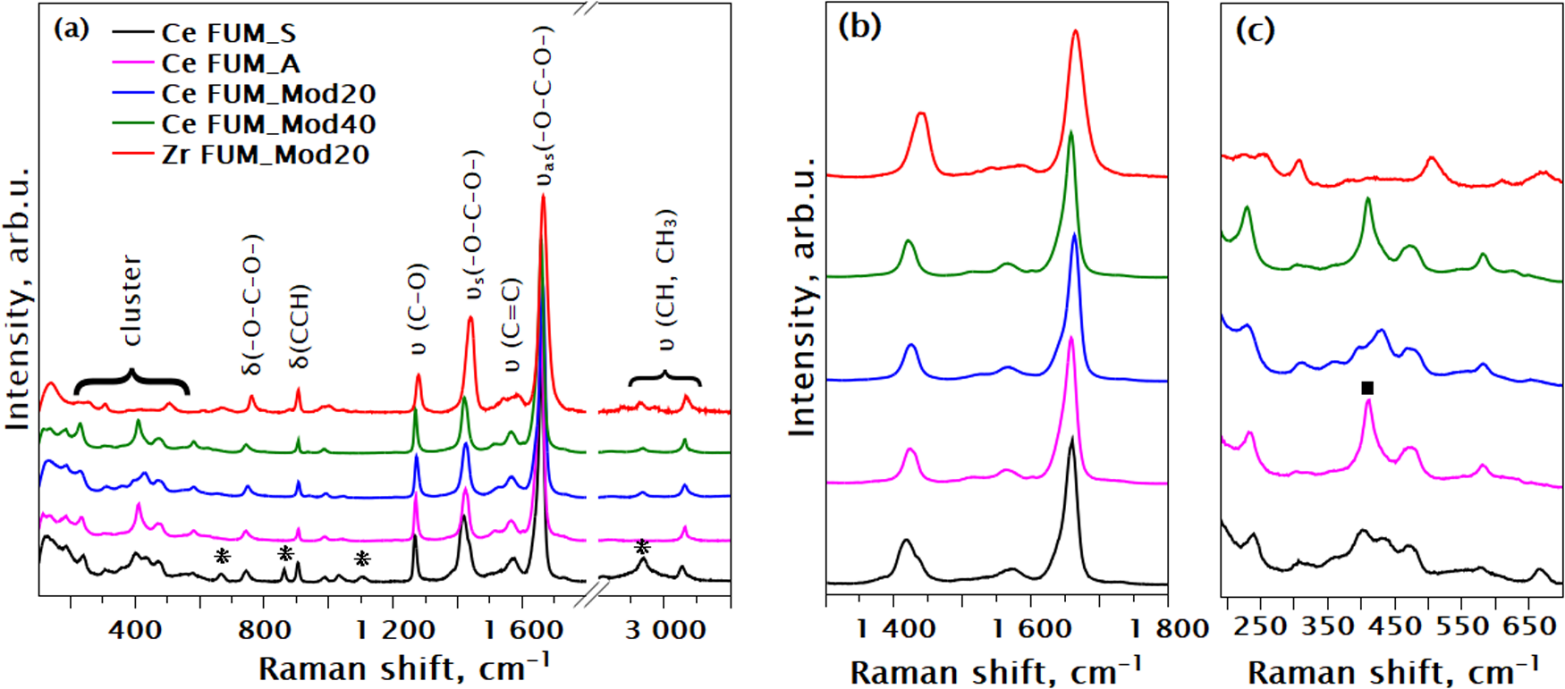
(a) Raman spectra of washed and dried MOFs measured with the 532 nm wavelength, normalized to maximum; υ, stretching vibration; δ, bending vibration; a and as, symmetric and asymmetric modes, respectively; (b) close-up of Me-linker bond region; (c) a close-up of Me–O region of the Raman spectra.

Figure 11 illustrates the Raman spectra of the final washed and dried MOF powders. As we are working with solids, there are more and sharper Raman features visible, compared to those in solution. A great similarity in features is evident among all the final MOF products. However, there are noticeable differences in the position of the characteristic −O–C–O– bond of the cluster-fumarate coordination in Ce FUM_Mod40 and Zr FUM_Mod20 from those of other Ce-fumarate samples (Figure 11b). The first sample shows a redshift of 4 cm ^**–**1^ in the position of the 1660 cm^**–**1^ peak in the remaining Ce-products. In Zr-fumarate, this mode is on the contrary blueshifted to 1666 cm^**–**1^. The shift of the Zr-based MOF to higher 2θ angles in the low-angle region of the XRD pattern aligns well with the position of the Raman shift observed for the Zr-fumarate −O–C–O– mode compared to that of its Ce-fumarate counterpart (Figure 11b). This is because, in general, the Raman shift of a bond exhibits an inverse proportionality to its bond length (and in this case, the unit cell parameter).

We also find significant differences in the M–O Raman region not only between the Zr- and Ce-oxo-clusters but between the Raman spectra of the Ce-samples (Figure 11c). Another difference is extra peaks in the Ce FUM_S sample compared with others (marked with an asterisk in Figure 11a). These correlate with the presence of residual acetone (CH_3_ stretching at 2937 cm^****–****1^) and Ce moiety (at 1033 cm^****–****1^) in the pores of the Ce FUM_S. Proton NMR spectroscopy and comparison of the Ce L_3_-edge EXAFS spectra of Ce FUM_S and Ce FUM_A further corroborate these findings (see Figures S5 and S6 in the Supporting Information).

Assigning specific vibrational modes to Raman peaks of MOFs is challenging, since the vibrations of most molecular groups are coupled. Many peaks can be closely spaced in frequency; disorders or defects in the metal cluster can activate otherwise symmetry-forbidden modes. Nevertheless, the comparison of the spectra of the two isostructural samples with different metal clusters provides immense help in distinguishing between the common modes of the linker and the specific features due to the metal oxo-clusters. Thus, we provide tentative assignment of the Raman spectra in Table 3 comparing our results with literature data.

**Table 3:** Assignment of Raman Spectra of the MOF Reaction Products

Raman shift, cm^**–**1^	moiety	assignment with references
3065	linker	C–H asym stretching^28^
2937	modulator/washing agent	CH_3_ stretching^42^
1666/1660 (Zr/Ce)	linker	–O–C–O– asym. stretching^28^
1573	linker	C=C stretching^28^
1441/1425 (Zr/Ce)	linker	–O–C–O– sym stretching^28,58^
1280/1270 (Zr/Ce)	linker	C–O stretching^59^
990	linker	out of plane CCH^28^
910	linker	out of plane CCH^28^
760/747 (Zr/Ce)	linker	–O–C–O– out of plane^28^
629 Ce	cluster+linker	O–H bending + C=O–O bending^58^
583 Ce	cluster	Ce–O^60^
505 Zr	cluster	Zr–O^56^
475 Ce	cluster+linker	Ce–O breathing sym^40,61^
430 Ce	cluster+capping moiety	Ce–O breathing sym^40,61^
412 Ce	cluster+capping moiety	Ce–O(−C–O−) sym stretching^58,61^
357 Ce	cluster+linker	Ce–O breathing asym^58^
300 Zr	cluster	Zr–O^56^
230–150	cluster+linker	complex modes Me–O(−O–C–O−)^58^

Figure S8 in the Supporting Information presents the SEM images of all synthesized samples. A comparison of the Ce-based fumarate MOFs reveals a consistent crystal grains and morphology across all samples, apart from Ce FUM_S. Notably, Ce FUM_S exhibits significantly smaller crystal grains compared to the other Ce-based MOFs. This observation aligns well with the broader small-angle peaks observed in the PXRD patterns for Ce FUM_S (Figure 10b), suggesting the less crystalline nature of this sample. In contrast, the Zr FUM_Mod20 sample displays increased crystallinity and larger crystal grain size, consistent with the sharper small-angle peaks present in its PXRD pattern (Figure 10b).

Considering all the observations, modulated synthesis samples (Ce_FUM_Mod20, Ce_FUM_Mod40, and Zr_FUM_Mod20) may possess a combination of the missing linker and cluster defects. This could also explain the expanded unit cell observed in Ce_FUM_Mod40 through PXRD peak positions compared to other Ce-based fumarate MOFs.

Our studies demonstrate that established modulation mechanisms do not play a significant role in the synthesis of Ce-fumarate in water in the investigated concentrations (20–40 equiv) and with the given precursor, as evidenced by the negligible effect observed upon addition of acetic acid as a modulator. Combining the fumarate linker and CAN in the reaction vessel leads to rapid formation of MOF and crystallization. Zr-fumarate MOF synthesis follows a distinct pathway. An intermediate Zr phase forms during the initial induction period, most probably coordinated by acetic acid, followed by the formation of oxo-clusters and the rearranging of the coordination to the fumarate with the subsequent MOF crystallization after 25 min into the reaction and at 95 °C.

Figure S12 compares the kinetic curves obtained from in situ Raman spectroscopy and synchrotron PXRD. It is clear that conventional in situ PXRD can be effectively replaced by the simpler in situ Raman technique for monitoring MOF formation and constructing kinetic profiles. We propose that Raman spectroscopy offers advantages in this context, as it can detect noncrystalline and liquid products in addition to plotting kinetic curves. This capability has enabled us to better understand and describe potential reaction pathways, which is not possible with in situ PXRD.

## 5 Conclusions

This study presents the first application of in situ Raman spectroscopy to monitor the water-based synthesis of Zr- and Ce-fumarate MOFs. Raman spectroscopy’s sensitivity to both crystalline and amorphous phases enabled direct observation of the reaction pathway and identification of acetic acid’s modulatory role. We have shown that acetic acid plays a negligible role, if any, as a modulator in Ce-fumarate MOF synthesis. Upon combining the fumarate linker and CAN in the reaction vessel, Ce oxo-clusters form promptly in parallel with coupling to the fumaric linkers, which initiates MOF crystallization immediately. The parallel formation of Ce oxo-clusters and their coordination to the fumarate linker were evident from the real-time development of the corresponding Raman features of Ce oxo-cluster and linker–metal coordination. Zr-fumarate synthesis follows a slower kinetics that allows us to distinguish the steps where preliminary Zr–O clusters are formed possibly coordinated by acetate in an alternative to bridged or bidentate coordination. Afterward, the formation of Zr-oxo-clusters, MOF building blocks, comes at the same time when the coordination is changed to the fumarate linkers.

Reaction kinetics based on the integrated peak area of the moieties of interest can be studied provided a suitable remedy for the background and temperature effects in the data. The most reliable kinetics data were obtained for the room-temperature syntheses of Ce-fumarates with negligible variations in the background. The increasing intensity of the 1666 cm^–1^ peak in the Raman data, responsible for the linker–metal coordination, corresponded to the crystallite growth until the precipitation from the measurement volumes occurred, which was observed as the decline in the respective intensities. This conclusion was backed up by the in situ PXRD data.

Importantly, our findings establish Raman spectroscopy as a viable tool for real-time monitoring of MOF synthesis monitoring. The technique can offer kinetic data comparable to those of the in situ PXRD method. The latter, however, cannot assess the amorphous and liquid reaction products in contrast to the Raman scattering analysis. Additionally, access to the large-scale synchrotron facilities is usually required for the in situ PXRD measurements, whereas Raman experiments can be realized in a home laboratory or at an industrial site. The completion of fumarate MOF formation can be conveniently tracked by monitoring the saturation intensity of the characteristic Raman peak at 1666 cm**^–^**^1^ responsible for the metal-fumarate coordination. We have also demonstrated a versatile reactor that can be utilized for both Raman and PXRD studies, providing direct comparison between the information obtained from both the experiments.

## Supporting Information Available

Details on synthesis procedures and the in situ reactor, in situ Raman data of water solutions of Ce- and Zr-precursors, details of Raman peak fitting, and additional characterization of the reactions and the reaction products (PDF)
