# Supplementary material for: Probing Ce- and Zr-Fumarate Metal–Organic Framework Formation in Aqueous Solutions with In Situ Raman Spectroscopy and Synchrotron X-ray Diffraction
Source: ACS Omega. 2024 Oct 21;9(44):44321–35. doi: 10.1021/acsomega.4c05125 (PMC11541789; doi:10.1021/acsomega.4c05125)
Supplement: Supplementary file 1 — ao4c05125_si_001.pdf [file ao4c05125_si_001.pdf]

Supporting Information

Probing Ce- and Zr- Fumarate MOF Formation in Aqueous Solutions with In-situ Raman Spectroscopy and Synchrotron X-ray Diffraction

Sofiia Bercha[1], Simmy Rathod[2], Olena Zavorotynska[1*], Sachin Maruti Chavan[2*]

[1] *Department of Mathematics and Physics, University of Stavanger, P.O. Box 8600, NO-4036, Stavanger, Norway*

[2] *Department of Chemistry, Bioscience and Environmental Engineering, University of Stavanger, P.O. Box 8600, NO-4036, Stavanger, Norway*

*Corresponding authors: olena.zavorotynska@uis.no, sachin.m.chavan@uis.no.

Contents

1. Materials used for the in-situ synthesis and illustration of the R*T* synthesis reactor......3

2. Following the Ce and Zr cluster formation with in-situ Raman 3

3. Impact of temperature on Raman scattering intensity and raw Raman data of the H*T* synthesis 4

4. Proton NMR and XAS Ce L3-edge comparison..6

5. Determining defects in MOFs using TGA analysis...7

6. SEM images of the washed and dried MOF powders..8

7. Pore size distribution, derived from the N_2 sorption at 77 K...9

8. Raman spectra changes over 2 months period. 10

9. In-situ Raman and in-situ XRD MOF comparison 11

10. Investigating coordination mode in MOFs using Raman spectroscopy........................11

11. Kinetic curves comparison 12

References 13

1. Materials used for the in-situ synthesis and illustration of the R*T* synthesis reactor

Table S1. Reagents used in the MOF synthesis

Compound	Reagent quantities					
	Cerium ammonium nitrade (CAN)	Zirconium Sulfate Tetrahydrate	Fumaric acid	Acetic acid	Water	Stoichiometry, eqv. (Me:FA:MOD:H_2O)
Ce FUM_S	438.6 mg	n/a	92.8 mg	n/a	4 ml	1 : 1 : 0 : 280
Ce FUM_A	438.6 mg	n/a	92.8 mg	n/a	4 ml	1 : 1 : 0 : 280
Ce FUM_Mod20	438.6 mg	n/a	92.8 mg	0.914ml	4 ml	1 : 1 : 20 : 280
Ce FUM_Mod40	438.6 mg	n/a	92.8 mg	1.828 ml	4 ml	1 : 1 : 40 : 280
Zr FUM_Mod20	n/a	305 mg	374 mg	1.23 ml	4 ml	1: 3 : 20 : 212

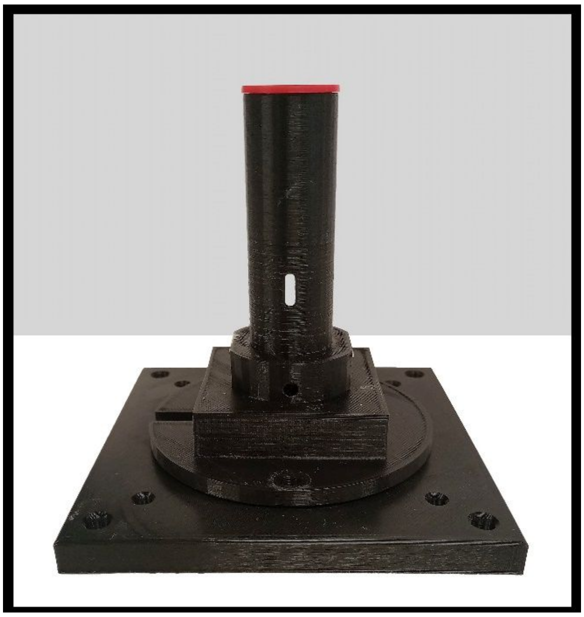

Fig. S1. 3D printed plastic R*T* in-situ reactor.

2. Following the Ce and Zr cluster formation with in-situ Raman

Figure S2 shows in-situ Raman following Ce and Zr cluster formation in the indicated times (and temperatures in case of Zr cluster). The reactions were carried out in identical conditions to the respective MOF-syntheses except for the absent fumarate linker. The integrated area of the characteristic acetic acid υ(C–C) band at 890 cm^{-1} remains relatively constant in all the modulated synthesis cluster formation analogues, as illustrated in Fig. S2(d). Across all the spectra, no additional peaks indicative of Ce- or Zr-acetate cluster formation were observed[1,2]. The evolution of the spectra with time is solely characterized by the appearance and growth of peaks associated with metal salt hydrolysis, specifically nitric acid for Ce (1050 cm^{-1}) and sulfuric acid for Zr precursor hydrolysis (1055 cm^{-1}). In the Raman spectrum of Ce cluster formation with 20 eqv. of acetic acid modulator (Fig. S2(b)), a weak feature is observed at 945

cm⁻¹ (indicated by the arrow). While this could potentially be assigned to the strongest Raman Ce-acetate mode,[1] its very low intensity makes such an assignment inconclusive.

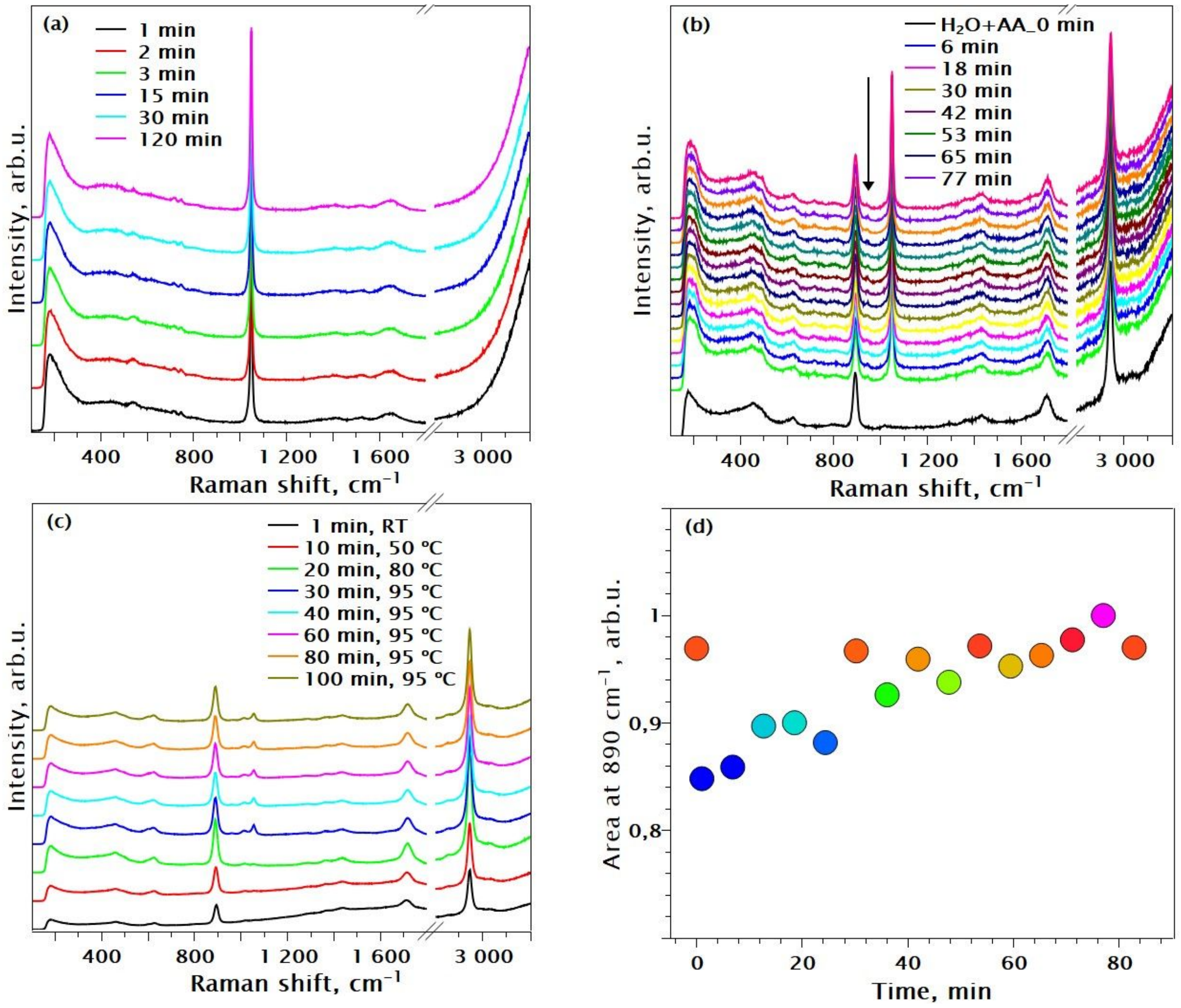

Fig. S2. (a) in-situ Raman following the Ce cluster formation (no linker) at RT; (b) in-situ Raman following the Ce cluster formation with 20 eqv. acetic acid modulator (no linker) at RT; (c) in-situ Raman following the Zr cluster formation with 20 eqv acetic acid modulator at HT; (d) fitted area of the acetic acid Raman band at 890 cm^{-1} during Ce cluster formation with 20 eqv acetic acid vs. time of reaction, data normalised by maximum for easier comparison

3. Impact of temperature on Raman scattering intensity and raw Raman data of the H*T* synthesis.

The decrease in Raman band intensity with rising temperature is a well-established phenomenon attributed to various factors, with phonon anharmonicity being the primary contributor. Since we are analysing H*T* Zr-fumarate MOF synthesis with Raman scattering, to perform adequate analysis of the Raman bands for kinetic investigation we implemented intensity normalization with respect to room temperature (R*T*), following the approach employed by Embrechts et al.[3]

The impact of temperature on the band intensity was investigated by integrating the acetic acid band area at 890 cm^{-1} during the reactor's cooling process after the Zr FUM_Mod20 synthesis. A slight negative linear correlation was observed between temperature and Raman band

intensity. The normalized band area as a function of temperature (°C), with an area of 1.0 set for a temperature of 25°C, follows the relationship y = -1.7 × 10^{-3}x + 1.066, as shown in Fig. S3. This equation exhibits an R^2 value of 0.86, indicating a strong correlation between temperature and band intensity. Intensities of the Raman scattering spectra were normalised for the H*T* Zr-fumarate MOF synthesis and H*T* Zr cluster formation were normalised accordingly.

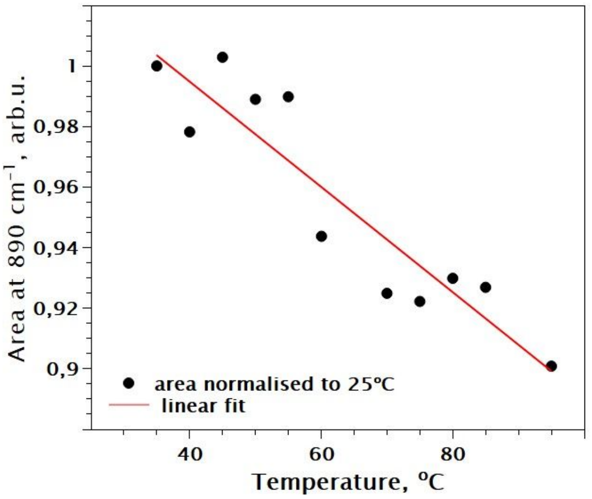

Fig. S3. Raman signal of the acetic acid vs. temperature. Linear fit was used to investigate the dependence of Raman signal intensity on temperature and calibration of the H*T* synthesis spectra accordingly.

Fig. S4 presents additional fluctuations in the background signal evident in the raw data acquired during the Zr-fumarate H*T* synthesis. These fluctuations are likely attributable to the electronic polarizability of ions such as SO_4^{2-}, acetate, and water molecules within the reaction vessel. When light interacts with these species, it undergoes inelastic scattering, resulting in a diffuse and featureless background in the Raman spectrum. Notably, this effect manifests prominently as a broad feature around the water band at approximately 1650 cm^{-1} within the mid-region of the spectrum, which diminishes as the reactor temperature surpasses 95 °C.

Substantial alterations observed in the Me-O cluster region (100-600 cm^{-1}) serve as a distinct indicator of zirconium oxo-cluster formation upon reaching 95 °C (illustrated by the pink line in Fig. S4).

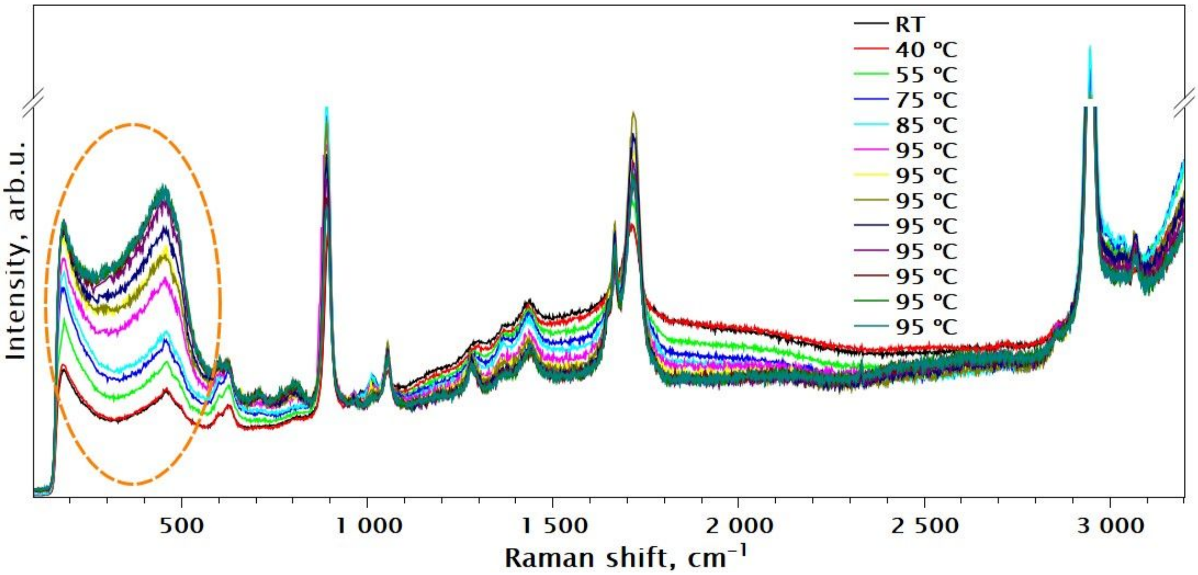

Fig. S4. Raw data for the Zr-fumarate H*T* synthesis at indicated temperatures. Illustration of background changes in the mid-region of spectra. Orange ellipse emphasizes the changes in the Me-O region indicating the formation of Zr-oxocluster.

4. Proton NMR and XAS Ce L3-edge comparison

Fig. S5 shows the proton NMR of the washed and dried samples. As expected, Ce FUM_A has no signs of acetic acid. The Zr FUM_Mod20 also has no acetic acid present but shows negligible amounts of ethanol originating from washing. Ce FUM_Mod20 and Ce FUM_Mod40 show the ratio between fumaric and acetic acids of approximately 1:0.16. We tentatively assign the 2.1 ppm peak of the Ce FUM_S sample to acetone coming from washing the sample, however, the 8.4 ppm feature is coming from the moieties stuck inside the pores of this sample. We propose that the gradual addition of the Ce salt and immediate formation of cerium-fumarate bonds led to the formation of pores around existing Ce moieties in the reaction vessel, effectively encapsulating them. The observed atypical weight loss noted during TGA analysis (Fig. S7), coupled with the emergence of supplementary Raman bands in this sample, supports our hypothesis. Additionally, this idea finds further backing from a comparison of the X-ray absorption spectroscopy (XAS) Ce L_3-edge spectra of the Ce FUM_S and Ce FUM_A samples (Fig. S6). Notably, the Ce FUM_S sample exhibits an additional phase of cerium, as evidenced by lower E_0 value, compared to the Ce FUM_A sample.

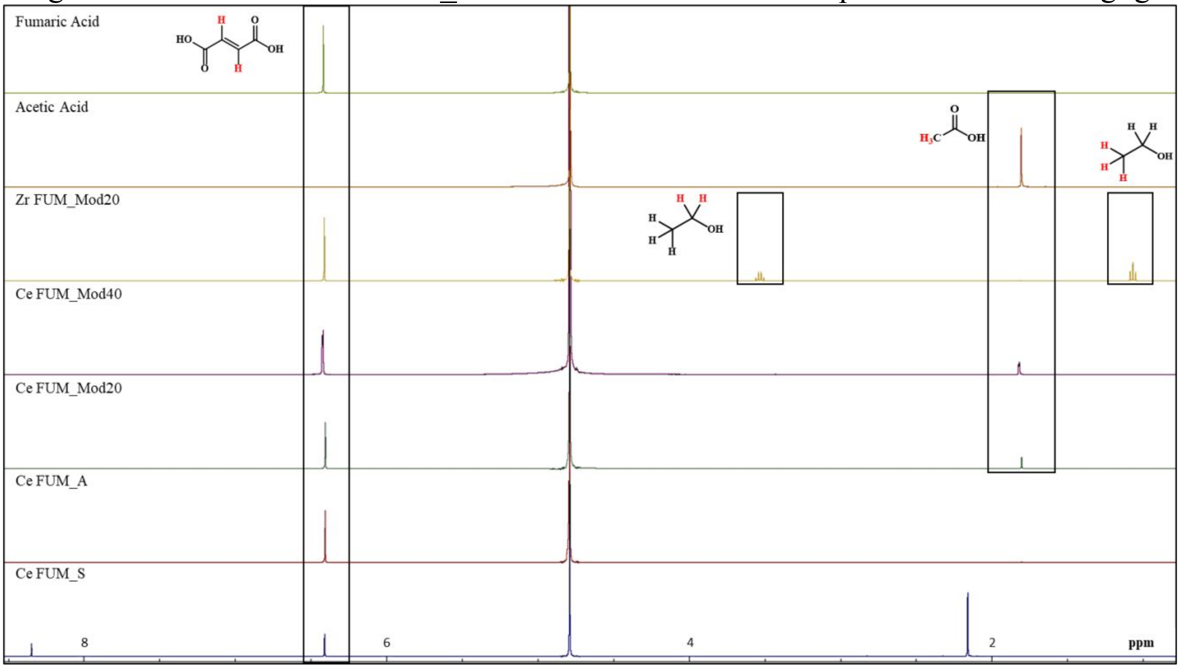

Fig. S5. Proton NMR of all the washed and dried MOFs. Upper panels show the proton NMR of fumaric acid and acetic acid for comparison.

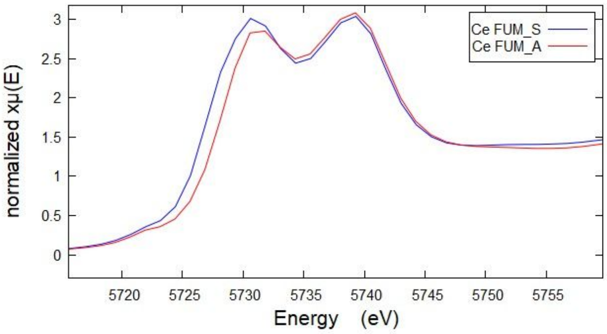

Fig. S6. XAS Ce L_3-edge of Ce FUM_S and Ce FUM_A comparison

5. Determining defects in MOFs using TGA analysis

Conventional way of determining the defects in UiO-66 analogue MOFs using the TGA analysis has been established in the MOF community, and the excellent discussion on this topic is provided in the support information of the work by Gibbons et al. [4]

**Table S2**. Calculation of the ideal MOFs weight loss percentage			
	Ideal Structure	Molecular Weight (g/mol)	Percentage of Final Product Weight
Starting Zr Fumarate MOF	$Zr_6O_4(OH)_4(C_4H_2O_4)_6$	1363	186%
First TGA transformation of Zr Fumarate MOF	$Zr_6O_6(C_4H_2O_4)_6$	1327	**181%**
Final product after TGA analysis	$6ZrO_2$	731	100%
Starting Ce Fumarate MOF	$Ce_6O_4(OH)_4(C_4H_2O_4)_6$	1657	160%
First TGA transformation of Ce Fumarate MOF	$Ce_6O_6(C_4H_2O_4)_6$	1621	**157%**
Final product after TGA analysis	$6CeO_2$	1032	100%

Here we follow the procedure implementing it to the Ce-fumarate MOF samples, as our Zr FUM_Mod20 sample is not showing deviation from the ideal structure weight loss (refer to Fig. S7 and Table S2). To determine the number of missing linkers in the MOF, the material is heated to temperatures beyond 550 °C under air until it completely decomposes into CeO_2. The weight loss between the final metal oxide and the plateau directly preceding it is attributed to the loss of MOF linkers. The total weight loss of organic material is then divided by the weight loss per linker to calculate the number of linkers in the MOF. For a pristine, defect-free Ce fumarate MOF sample, the weight directly preceding organic decomposition is 157%, the final weight is normalized to 100%, and the number of linkers is 6, resulting in a weight loss per linker of 9.5%. Using this value, we have calculated the percentage of defect level in our actual Ce-fumarate MOF samples. It resulted in very similar 16% of linker defects for the Ce FUM_A, Ce FUM_Mod20 and Ce FUM_Mod40. This procedure couldn't be adequately implemented for the Ce FUM_S samples, due to extra moieties stuck in the pores, mentioned before.

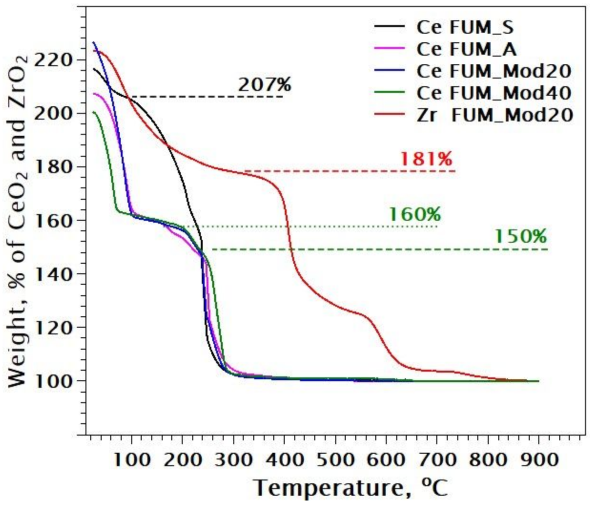

Fig. S7. TGA weight loss profiles of the MOF samples. Dashed lines and persentages represent the weight plateau right before the decomposition of the MOF structure. Green dotted line represent the weight plateau of Ce-fumarate MOF samples before loosing caping acetic acid or water moeities.

.

6. SEM images of the washed and dried MOF powders

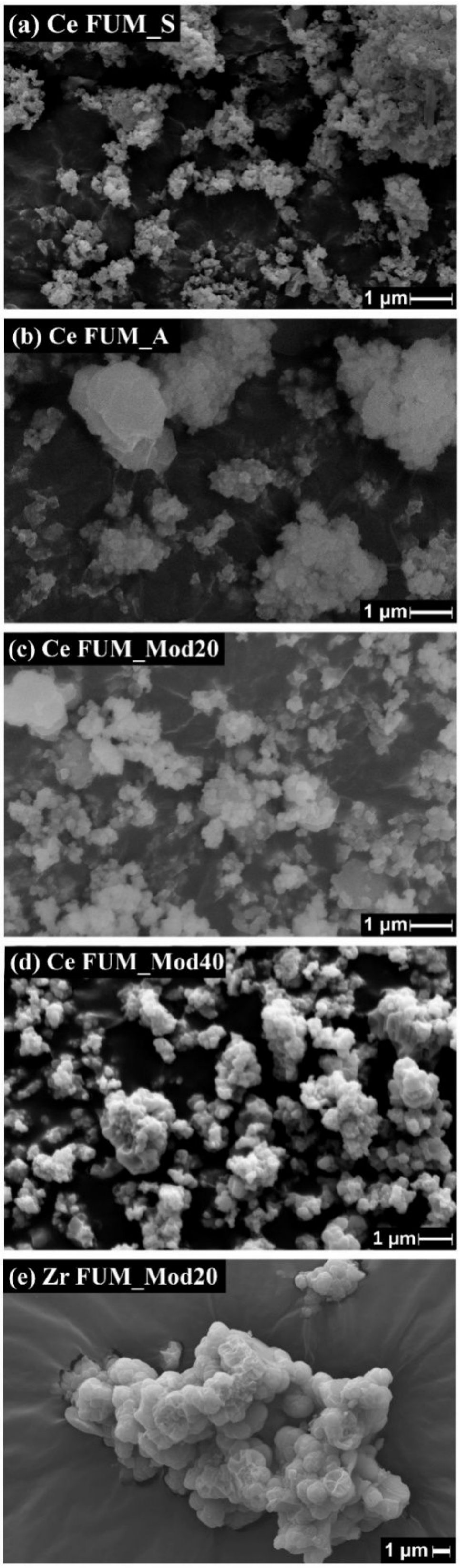

Fig. S8. SEM images of the washed and dried MOF powders

7. Pore size distribution, derived from the N_2 sorption at 77 K

Pore size distribution was calculated using DFT method implemented in the Micromeritics TriStar II system software.

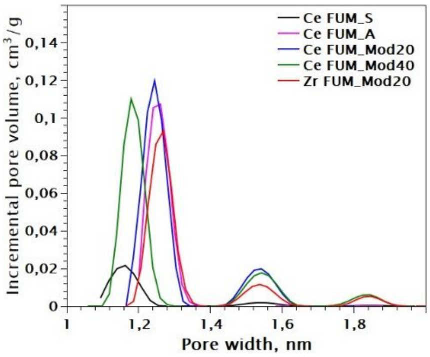

Fig. S9. Pore size distribution of Ce- and Zr- fumarate MOF samples

8. Raman spectra changes over 2 months period.

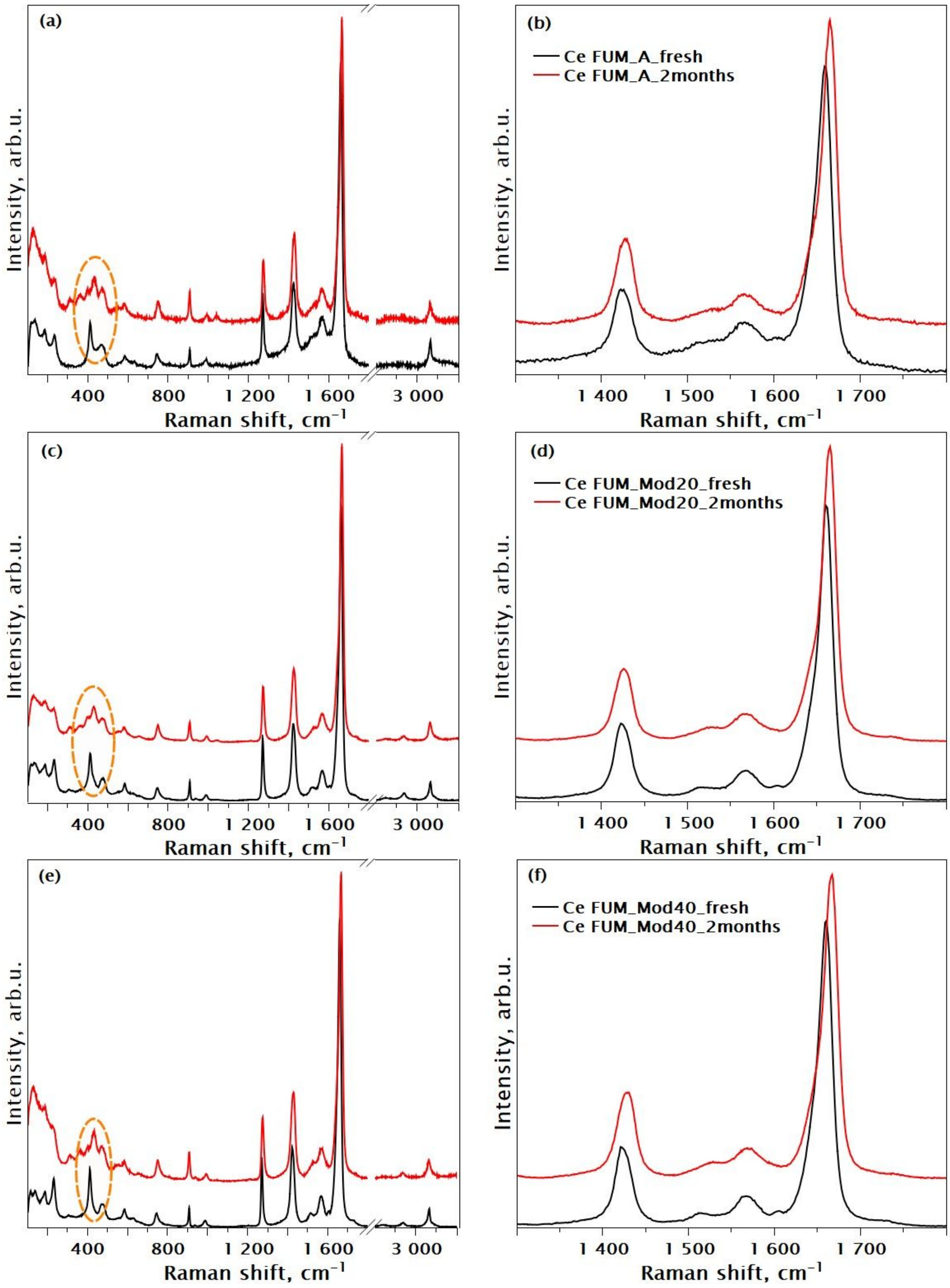

Fig. S10 (a) Ce FUM_S; (b) close-up of the metal-fumarate bond region of (a); (c) Ce FUM_Mod20; (d) close-up of the metal-fumarate bond region of (c); (e) Ce FUM_Mod40 R; (f) close-up of the metal-fumarate bond region of (e).

Distinguishable differences in the Raman intensities of the Ce-based MOFs in the Me-O region and shifts of the peaks in the mid-wavelength region were observed when spectra were measured after 2 months period of storing in ambient atmosphere (Fig. S10). The Ce FUM_S and Zr FUM_Mod20 didn't show significant differences, so their spectra are not shown here.

9. In-situ Raman and in-situ XRD MOF comparison

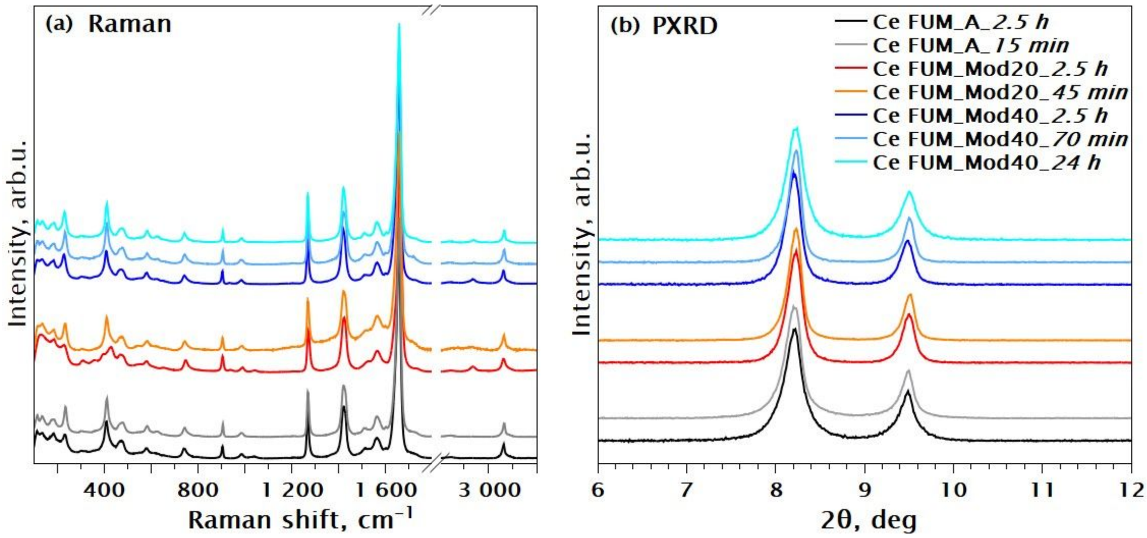

Fig. S11. Comparison of Raman spectra (a) and XRD patterns (b) of the washed and dried Ce-based MOFs obtained during in-situ Raman analysis and in-situ XRD analysis. A synthesis time of 2.5 hours was used for all in-situ Raman investigations. The times for in-situ PXRD investigations are noted for each sample. Spectra of 24 h synthesis (decomposition trial) of the Ce FUM_Mod40 is also included.

10. Investigating coordination mode in MOFs using Raman spectroscopy

In a comprehensive review by Hadjiivanov et al.,[5] the application of vibrational spectroscopies for characterizing MOFs is explored. They highlight the utility of the splitting between symmetrical and asymmetrical stretching vibrations of the carboxylate (–O–C–O–) groups in MOFs. This splitting, denoted as $\Delta\nu_{exp}$, provides valuable information about the coordination mode of the carboxylate ligands within the framework structure. It is suggested high $\Delta\nu_{exp}$ values (>180 cm^{-1}) signify monodentate carboxylate coordination, while values <120 cm^{-1} indicate chelating or bridging configurations. Values between 160-170 cm^{-1} could point towards ionic COO^{-} bridging, or H-bonded monodentate coordination.

In our Zr-fumarate and Ce-fumarate MOFs, $\Delta\nu_{exp}$ values were 225 cm^{-1} and 235 cm^{-1}, respectively and fall within the monodentate range. Notably, these values remained consistent during in-situ characterization and with washed and dried MOF powders.

However, despite the high $\Delta\nu_{exp}$ values, combined evidence provided by all characterization methods revealed a bridged bidentate coordination mode for the metal-linker interaction in both the synthesized MOFs and during their formation.

It is important to note that the above correlations are empirical and should not be used for definitive conclusions. For the UiO-66 type MOFs well established $\Delta\nu_{exp}$ is exciding 200 cm^{-1} and the metal-linker interaction is bridged bidentate.[6]

11. Kinetic curves comparison

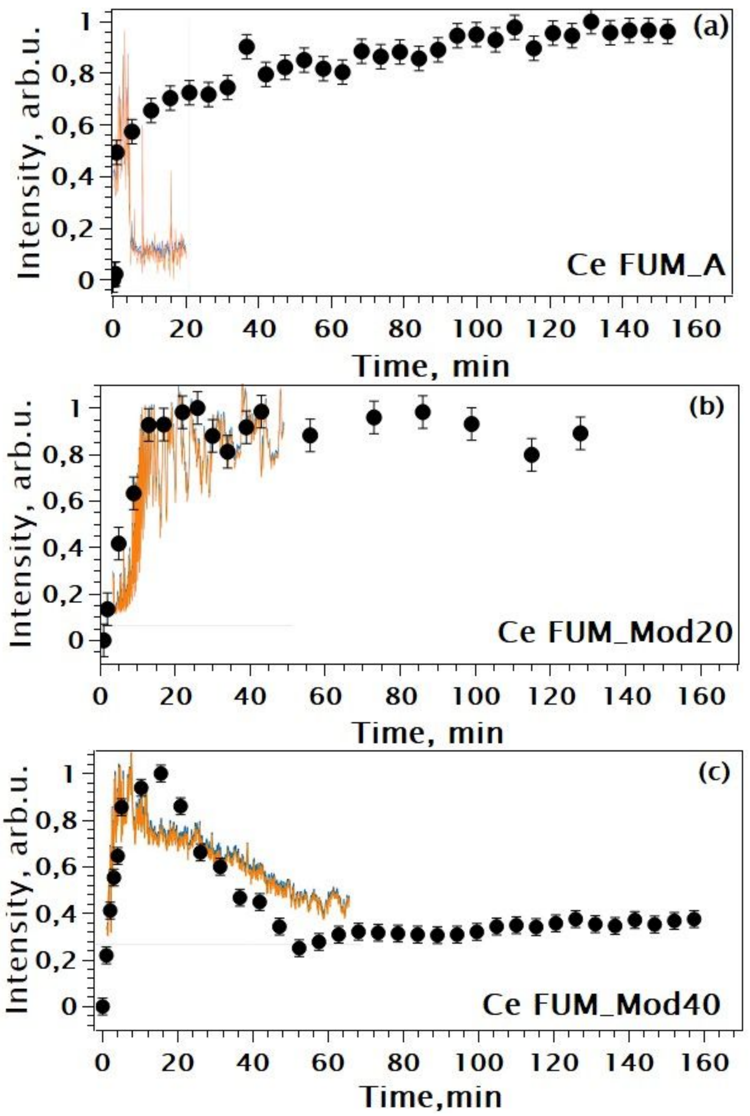

Fig. S12. Comparison of the kinetic curves obtained with in-situ Raman spectroscopy (black circles) and in-situ synchrotron PXRD (orange and blue lines) for (a) Ce FUM_A, (b) Ce FUM_Mod20 and (c) Ce FUM_Mod40 samples.

References

(1) *Ce III acetate Raman*. https://www.chemicalbook.com/Spectrum/CB9471476_Raman_0.jpg

(2) Straughan, B. P.; Moore, W.; Mclaughlin, R. Structural Investigations of Zirconium Tetra-Acetate and the Group IVB Tetra-Acetates. *Spectrochim. Acta A*, **1986**, 42, 451-456.

(3) Embrechts, H.; Kriesten, M.; Hoffmann, K.; Peukert, W.; Hartmann, M.; Distaso, M. Elucidation of the Formation Mechanism of Metal-Organic Frameworks via in-Situ Raman and FTIR Spectroscopy under Solvothermal Conditions. *J Phys Chem C* **2018**, *122* (23), 12267–12278.

(4) Gibbons, B.; Bartlett, E. C.; Cai, M.; Yang, X.; Johnson, E. M.; Morris, A. J. Defect Level and Particle Size Effects on the Hydrolysis of Chemical Warfare Agent Simulant by UiO-66. *Inorg Chem*, **2021**, 60, 16378–16387.

(5) Hadjiivanov, K. I.; Panayotov, D. A.; Mihaylov, M. Y.; Ivanova, E. Z.; Chakarova, K. K.; Andonova, S. M.; Drenchev, N. L. Power of Infrared and Raman Spectroscopies to Characterize Metal-Organic Frameworks and Investigate Their Interaction with Guest Molecules. *Chem Rev*, **2021**, 121, 1286–1424.

(6) Valenzano, L.; Civalleri, B.; Chavan, S.; Bordiga, S.; Nilsen, M. H.; Jakobsen, S.; Lillerud, K. P.; Lamberti, C. Disclosing the Complex Structure of UiO-66 Metal Organic Framework: A Synergic Combination of Experiment and Theory. *Chem Mat* **2011**, *23* (7), 1700–1718.
